# A Photo‐responsive Small‐Molecule Approach for the Opto‐epigenetic Modulation of DNA Methylation

**DOI:** 10.1002/anie.201901139

**Published:** 2019-04-12

**Authors:** Ha Phuong Nguyen, Sabrina Stewart, Mikiembo N. Kukwikila, Sioned Fôn Jones, Daniel Offenbartl‐Stiegert, Shiqing Mao, Shankar Balasubramanian, Stephan Beck, Stefan Howorka

**Affiliations:** ^1^ Department of Chemistry Institute for Structural and Molecular Biology University College London 20 Gordon Street London WC1H 0AJ UK; ^2^ UCL Cancer Institute London UK; ^3^ Department of Chemistry University of Cambridge Lensfield Road Cambridge UK; ^4^ Cancer Research (UK) Cambridge Institute University of Cambridge Robinson Way Cambridge UK

**Keywords:** cytosine, DNA, epigenetics, methylation, photo-caging

## Abstract

Controlling the functional dynamics of DNA within living cells is essential in biomedical research. Epigenetic modifications such as DNA methylation play a key role in this endeavour. DNA methylation can be controlled by genetic means. Yet there are few chemical tools available for the spatial and temporal modulation of this modification. Herein, we present a small‐molecule approach to modulate DNA methylation with light. The strategy uses a photo‐tuneable version of a clinically used drug (5‐aza‐2′‐deoxycytidine) to alter the catalytic activity of DNA methyltransferases, the enzymes that methylate DNA. After uptake by cells, the photo‐regulated molecule can be light‐controlled to reduce genome‐wide DNA methylation levels in proliferating cells. The chemical tool complements genetic, biochemical, and pharmacological approaches to study the role of DNA methylation in biology and medicine.

## Graphical abstract

**A small‐molecule approach** for the modulation of DNA methylation with light is presented. The strategy uses a photo‐tunable version of a clinically used drug (5‐aza‐2′‐deoxycytidine) to alter the catalytic activity of DNA methyltransferases. After uptake by cells, the photo‐regulated molecule can be light‐controlled to reduce genome‐wide DNA methylation levels in proliferating cells.

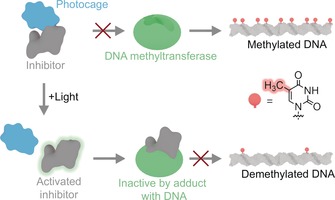

The methylation of DNA at position 5 of cytosine residues is chemically a very simple but biologically one of the most important modifications of DNA. It influences many biological processes in humans such as the regulation of cell function, cellular reprogramming, and organismal development.^1^, ^2^, ^3^, ^4^, ^5^, ^6^, ^7^ Biological effects of higher methylation levels at promoters are mediated by lowering the transcription of genes either by blocking the binding of transcription factors or by recruiting unique methyl‐recognizing proteins that lower gene expression. Altered levels of methylation are also associated with several diseases^8^, ^9^, ^10^, ^11^ including cancer.^8^, ^12^, ^13^, ^14^, ^15^, ^16^

Driven by the growing importance of DNA methylation in biomedical research, there is a strong interest to experimentally lower or increase methylation levels^17^, ^18^, ^19^, ^20^, ^21^, ^22^, ^23^ to study, for example, the role of epigenetic reprogramming in tissue development or regenerative medicine.^24^, ^25^ Optical control is of particular relevance given the high spatial and temporal resolution of light. Often, the approach is implemented with photosensitive small molecules of tuneable bioactivity.^26^, ^27^, ^28^, ^29^, ^30^, ^31^ These can be used without the need for genetic engineering of cells leading to powerful applications within cell biology.^32^ Yet, despite the importance of DNA methylation in biology, no light‐tuneable small‐molecule tool has been developed to manipulate methylation levels in cells.

Herein, we present a photo‐mediated small‐molecule strategy that modulates methylation in light‐exposed cells. At the approach's centre is an inhibitor that interferes with DNA methyltransferases (DNMTs), the enzymes responsible for DNA methylation^33^ including the maintenance DNA methyltransferase 1 (DNMT1).^34^ The inhibitor's bioactivity becomes tuneable with light by the chemical derivatization with a photocage. As schematically illustrated in Figure 1 a, the attached photocage renders the inhibitor biologically inactive. However, light exposure cleaves off the photocage to restore the original inhibitory effect (Figure 1 a). The photocaged molecule is hence expected to maintain methylation levels in the dark, while light should decrease methylation levels following replication of cells^35^ (Figure 1 a).

**Figure 1:**
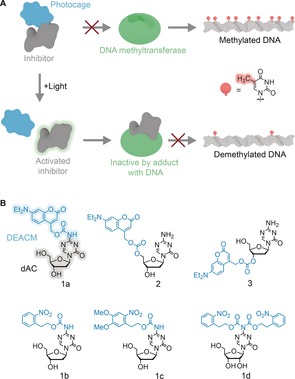
Photocaged derivatives of DNMT inhibitor 5‐aza‐2′‐deoxycytidine (dAC) designed to optically modulate the methylation of DNA. a) Scheme illustrating the principle of the photo‐caging approach. Photocaged inhibitor dAC is biologically inert and allows DNMT to maintain high methylation levels. Exposure to light removes the phototag to restore the inhibitory effect on DNMT to cause lowered DNA methylation with each round of DNA replication. b) Caged DNMT inhibitors N‐DEACMOC‐dAC (**1 a**), N‐NPEOC‐dAC (**1 b**), N‐DMNPEOC‐dAC (**1 c**), bis‐NPEOC‐AC (**1 d**), 5′‐DEACMOC‐dAC (**2**), and 3′‐DEACMOC‐dAC (**3**).

Our approach was implemented with DNMT inhibitor 5‐aza‐2′‐deoxycytidine (dAC, decitabine)^35^, ^36^ (Figure 1 b). The cytidine analogue is a clinically used drug for myelodysplastic syndromes^37^ and is being tested against leukemia and solid tumors^18^, ^38^ and as sensitizer for immunotherapies.^39^, ^40^ 5‐aza‐2′‐deoxycytidine is the best choice for the photocaging approach given its high inhibitory effect on DNMTs^41^ even though it is also known to undergo slow hydrolysis at the 5‐aza‐base ring.^42^ To exert its inhibitory effect after cellular uptake, dAC is phosphorylated by deoxycytidine kinase in a rate‐limiting step.^43^ Subsequent phosphorylations to triphosphate lead to the polymerase‐mediated incorporation into DNA^43^ in which the 5‐aza‐base ring forms a covalent adduct with DNMT. This adduct prevents methylation of DNA in replicating cells but also targets DNMT for proteosomal degradation.^44^ Given the tight fit inside the active site of deoxycytidine kinase (Supporting Information, Figure S1), we surmised that photocaging dAC would block the rate‐limiting step of phosphorylation and hence abolish inhibition of DNMT.

To optically control the activity of dAC, we attached a photocage to each possible coupling site within the nucleoside, the exocyclic NH_2_ group of the base and the 3′ and 5′ OH groups of the deoxyribose (Figure 1 b).^27^, ^31^ All three positions were modified as the resulting steric blockade was expected to hinder binding of dAC into the active site of deoxycytidine kinase (Figure S1). For the chemical derivatization, diethylaminocoumarinyl‐4‐methyl (DEACM) (Figure 1 b) was used given its high extinction coefficient (*ϵ*=16 000 m ^−1^ cm^−1^) and long absorption wavelength (*λ*=385 nm) that ensure biocompatibility by avoiding mutagenic irradiation at high intensity in the UV spectral region.

Three DEACM derivatives of dAC **1 a**, **2**, and **3** (Figure 1 b) were synthesized. In **1 a**, the photocage is attached through a carbamate bond to NH_2_, while the linkage in **2** and **3** is mediated through a carbonate to 5′ and 3′ OH, respectively (Figure 1 b). The synthetic routes to **1 a**, **2**, and **3** are described in the Supporting Methods.

Additional photocaged compounds were made to demonstrate that the synthetic route is generic. For example, synthesis of **1 b** and **1 c** carrying a nitrophenyl group on the exocyclic amine (Figure 1 b) showed that a chromophore other than DEACM can be attached to dAC. **1 b** and **1 c** also served as reference compounds for the spectroscopy analysis (see below). Similarly, preparation of nitrophenyl‐modified azacytidine **1 d** (Figure 1 b) showed that the clinically used ribonucleotide version of dAC can be equipped with a photocage (see Supporting Methods for synthetic routes of **1 b**–**d**).

DEACM‐dAC derivatives **1 a**, **2**, and **3** were examined to probe whether the spectroscopic properties are influenced by the chromophore's attachment site. All compounds exhibited strong absorption at a biocompatible wavelength of *λ*=365 nm (Figure 2 a, Table 1) with *ϵ* values close to that of unconjugated DEACM (*ϵ*=7000 m ^−1^ cm^−1^, Figure S2)^45^ implying minimal influence from the coupling to dAC. The data for compounds **1 b**–**d** showed similar results (Table 1, Figure S2).

**Figure 2:**
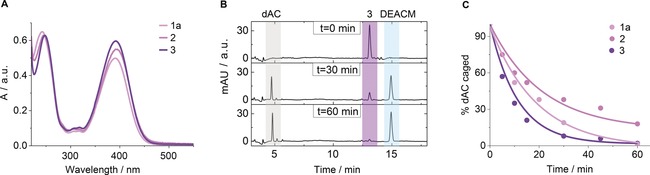
Spectroscopic and photochemical analysis of photocaged dAC versions **1 a**, **2** and **3**. a) UV/Vis absorption spectra of photocaged dAC compounds **1 a**, **2**, and **3** at 50 μm in DMSO/water (5:95). b) HPLC traces for the photodeprotection of **1 a**. The initial peak corresponding to caged **1 a** disappears upon irradiation at 365 nm to yield uncaged dAC and free DEACM‐OH. The rates for photo‐induced uncaging were determined by exposing the DEACM‐dAC conjugates to light of *λ*=365 nm at a moderate intensity of 145 μW cm^−2^ and at ambient temperature of 25 °C. c) Time course for photo‐induced uncaging of **1 a**, **2**, and **3** at *λ*=365 nm.

**Table 1:** Spectroscopic and photolytic properties of photocaged DNMT inhibitors.

	*λ*_max_^[a]^ [nm]	*ϵ*_*λ*max_^[b]^	*ϵ*_254_^[b]^	*ϵ*_365_^[b]^	*k* [s^−1^]^[c]^	*t*_1/2_ [min]	*Φ*_365_^[d]^	*ϵ*×*Φ*_365_^[e]^ [m ^−1^ cm^−1^]
**1 a**	391	10 000	11 000	7000	1.03×10^−3^	11	6.11×10^−2^	427
**1 b**	233	16 400	9000	200	8.33×10^−5^	139	4.93×10^−3^	0.99
					3.33×10^−4*^			
**1 c**	348	5000	10 400	4000	6.67×10^−5^	173	3.94×10^−3^	16
					1.00×10^−4*^			
**1 d**	260	14 800	12 100	460	n/a	n/a	n/a	n/a
**2**	395	11 000	11 400	7300	4.83×10^−4^	24	2.88×10^−2^	210
**3**	392	12 000	11 700	8100	1.50×10^−3^	8	8.84×10^−2^	716

[a] Wavelength of maximum absorption. [b] Molar absorptivities (m ^−1^ cm^−1^) at *λ*_max_, 254 nm, or 365 nm. [c] Deprotection rate constant for irradiation at 365 nm, or at 254 nm as indicated by *. [d] Quantum yield of uncaging at *λ*=365 nm. [e] Product of molar absorption coefficient and quantum yield of uncaging at *λ*=365 nm.

Uncaging efficiency, by contrast, was influenced by the site of dAC at which the chromophore was attached. The analysis (Figure 2 b) of compound **1 a** revealed a fast uncaging rate of *k*=1.03×10^−3^ s^−1^ equivalent to a 50 % recovery of dAC within a half‐life of *t*_1/2_=11 min (Figure 2 c) while **2** was slower (Figure 2 c and Figure S3), which is possibly due to a quenching interaction between the photocage and proximal triazine nucleobase. In support, **3** with DEACM at more distant 3′ OH to triazine had a fast photolysis with *t*_1/2_=8 min (Figure 2 b,c and Figure S3). The likely mechanism for uncaging is shown in Figure S4.

Successful uncoupling of the photocage from the nucleobase was also found for control nucleotides **1 b**–**d**. The spectroscopic and photolytic properties were in line with literature values for nitrophenyl (Table 1 and Figure S3). Nevertheless, the uncaging rates of **1 b**–**d** are too low for subsequent cell work. By comparison, compound **3** has a high absorption wavelength and the fastest photolysis.

Analysis of **3** determined its stability in the absence of light. Unmodified dAC is known to have a slightly reduced stability owing to hydrolysis at the 5‐aza‐base ring leading to a half‐life of 2200 min at 25 °C.^42^ By comparison, **3** had a half‐life of 690 min 25 °C, which reflects partial hydrolysis of the ring and the carbonate linkage to the photocage (Figure S5). This half‐life is almost 86‐fold longer than the half‐life for photo‐induced uncaging of **3** and 7‐fold longer than the subsequent incubation duration to cells. This means that after 1 h of light‐induced deprotection, only 3 % or less of compound **3** are still in the caged form. Dark instability is hence not compromising photo‐uncaging. Reflecting its adequate stability and fast deprotection rate under illumination, compound **3** was used for subsequent biological investigations.

To test whether methylation levels in cells can be controlled with light, **3** was added to hypermethylated human cancer cell lines SaOS2 and T24.^46^ Additional exposure of cells to light was expected to induce passive demethylation owing to photo‐uncaging of **3** and the resulting non‐methylation during DNA replication in dividing cells (Figure 3 b). Lack of illumination was anticipated to maintain methylation (Figure 3 a). Consequently, cells were incubated with 0.1 μm **3** and either illuminated for 1 h at 365 nm and 25 °C or kept in the dark at 25 °C. Treatment of cells with unmodified dAC served as a positive control for demethylation (Figure 3 c). After incubation with the small molecules, the medium was changed, cells were grown at 37 °C for 24 h, genomic DNA was isolated and enzymatically digested, and the nucleotide content was analysed by liquid chromatography coupled with tandem mass spectrometry (LC‐MS/MS).

**Figure 3:**
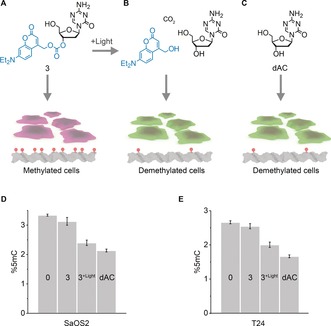
DEACMOC‐dAC **3** can be photo‐deprotected to re‐activate its inhibitory effect on DNMT and lower DNA methylation levels in cells. a–c) Schematic of cell treatment conditions and expected qualitative changes in DNA methylation levels. Treatment with **3** in the absence of light maintains high methylation levels (a), while illumination restores dAC activity to lower DNA methylation (b) to levels close to unmodified dAC (c). The concentration of **3** and dAC was 0.1 μm. Cells take up photocaged dAC at up to 4.5 μm within 1 h as shown using cell viability read‐out. d,e) Treatment‐dependent changes in methylation levels in SaOS2 (d) and T24 cell lines (e) for condition in (a–c) and 0 μm dAC under light exposure, as quantified by LC‐MS. DNA methylation levels (%5mC) are expressed as a percentage of total cytosines and analysed in biological triplicates.

Figure 3 d,e summarize the cellular levels of methylated C as percentage of the total cytosine pool for SaSO2 and T24 cells. Exposure to **3** without illumination maintained a high level of methylated DNA (Figure 3 d,e, **3**), thereby confirming that photocaged dAC was biologically inactive at the tested conditions. However, incubation with **3** and simultaneous exposure to light caused a drastic reduction in methylated DNA (Figure 3 d,e, **3**‐light) to a level almost identical to uncaged dAC (Figure 3 d,e, dAC). Control experiments in which cells were solely exposed to light in the absence of **3** did not affect methylation (Figure 3 d,e, 0, and Figure S6; 0 μm **3**). The data demonstrate that our strategy of light‐induced demethylation is successful; by photolysis of **3**, dAC's biological inhibition was reactivated to block DNA methyl transferases within cells. Our approach was also confirmed by demethylation at a concentration of 0.5 μm **3** (Figure S6). At 1.5 μm or higher, the compound leads to demethylation without light exposure, possibly because **3** is hydrolytically inactivated by enzymes.

Molecular analysis confirmed the proposed mechanism for **3**’s attainment of lower methylation levels in light‐exposed cells. First, an enzymatic assay established that the photocage in **3** interferes with deoxycytidine kinase activity. The kinase usually phosphorylates the 5′ OH of uncaged dAC^43^ after the compound is taken up by cells. However, the photocage attached to the 3′ OH of **3** prevents the compound's phosphorylation (Figure S7), most likely owing to sterically hindering access of **3** to the enzyme's active site (Figure S1). In addition, western blot analysis confirmed that uncaged **3** lowers methylation by decreasing levels of the DNA methyltransferase 1 (Figure S8). The amount of DNMT1 was reduced when cells were exposed to 0.1 μm **3** and light to liberate dAC. The inhibitor's mode of action is thought to involve its incorporation into DNA to form a covalent adduct with DNMT1,^43^ which prevents methylation of DNA in replicating cells but also targets DNMT for proteosomal degradation.^44^

This report has pioneered a light‐gated small‐molecule approach to regulate DNA methylation levels within cells. Thereby, our study breaks new ground in two areas. First, the photocaging of the DNA methyl transferase inhibitor achieves optically triggered DNA demethylation. Previously, there has not been any chemical tool available for light‐induced lowering of cellular methylation levels. Using genetically encoded epigenetic editing has previously yielded site‐specific DNA demethylation^17^ and methylation^23^ through TALE‐TET1 and Cas9‐DNMT fusion proteins, respectively. Light‐mediated regulation of site‐specific DNA methylation was attained with optogenetic protein pairs fused to DNMT and a locus‐targeting protein,^47^ similar to optically triggered demethylation with TET1.^48^ Photoactivation of a mutant dehydrogenase led to a decrease in 5‐hydroxymethylcytosine.^49^ The biological tools to target DNA methylation have been reviewed elsewhere.^22^ In a wider context, the non‐DNA epigenetic mark of histone methylation was modulated by optically controlled histone methyltransferases and histone deacetylases,^50^ and by a photoswitchable inhibitor of a deacetylase.^51^

Second, our study is the first to prepare photocaged dAC thereby providing rich chemical insight on an epigenetically important drug molecule as well as expanding the repertoire of caged nucleosides.^27^, ^31^, ^52^, ^53^ By generating a total of six dAC and ribonucleotide versions, we have uncovered information on efficient synthesis and on how the photocage's attachment site influences photolysis yield. Among the photocages tested, DEACM was found to be the best in terms of high wavelength absorption and photolytic efficiency, while carbonate or carbamate‐tethered nitrobenzyls **1 b**–**1 d** were not suitable, similar to previously tested ether‐based linkages. In practical terms, this insight could improve the future synthesis of photocaged versions of clinically tested dAC‐related drugs such as SGI‐110.^54^ Finally, dAC and related drugs could be modified with photoswitches that regulate bioactivity through photo‐isomerable conformation changes rather than photolysis.^26^, ^27^, ^28^, ^29^

The optically addressable DNMT inhibitor may be developed into a potentially valuable research tool for studying epigenetic mechanisms in health and disease. Areas of interest include regenerative medicine,^55^ developmental biology,^4^ development and progression of cancer,^56^ and the development of therapeutic routes^18^, ^38^, ^57^, ^58^, ^59^ to treat surface‐accessible tissues.^60^ Before realizing the potential, the photocaged nucleoside's bioavailability has to be successfully tested and its stability may have to be improved, for example, by replacing the carbonate tether with self‐immolating linkages.^61^, ^62^, ^63^ In the case of thicker tissues or organs, high‐wavelength photocages active in the optical window need to be devised. In conclusion, our photocaged DNMT inhibitor opens up exciting new avenues in basic and clinical research for epigenetics and also the synthesis of photo‐controlled molecules.

## Conflict of interest

The authors declare no conflict of interest. Sh.B. is an adviser and shareholder of Cambridge Epigentix Ltd.

## Supporting information

As a service to our authors and readers, this journal provides supporting information supplied by the authors. Such materials are peer reviewed and may be re‐organized for online delivery, but are not copy‐edited or typeset. Technical support issues arising from supporting information (other than missing files) should be addressed to the authors.

Supplementary
