# Supplementary material for: A Photo‐responsive Small‐Molecule Approach for the Opto‐epigenetic Modulation of DNA Methylation
Source: Angew Chem Int Ed Engl. 2019 Apr 12;58(20):6620–4. doi: 10.1002/anie.201901139 (PMC7027477; doi:10.1002/anie.201901139)
Supplement: Supplementary file 1 — Supplementary [file ANIE-58-6620-s001.pdf]

Angewandte Chemie
Eine Zeitschrift der Gesellschaft Deutscher Chemiker

Supporting Information

A Photo-responsive Small-Molecule Approach for the Opto-epigenetic Modulation of DNA Methylation

*Ha Phuong Nguyen, Sabrina Stewart, Mikiembo N. Kukwikila, Sioned Fôn Jones, Daniel Offenbartl-Stiegert, Shiqing Mao, Shankar Balasubramanian, Stephan Beck, and Stefan Howorka**

anie_201901139_sm_miscellaneous_information.pdf

Table of contents

1. Supporting methods 3

2. Procedures for the synthesis of compounds 1a-3 6

2.1.1. 7-(Diethylamino)-4-(hydroxymethyl)coumarin (5) 6

2.1.2. 7-(Diethylamino)-4-(hydroxymethyl)coumarin chloroformate (6) 7

2.1.3. 2′-Deoxy-4-*N*-[mono-7-(diethylamino)-2-oxochroman-4-yl)methoxy carbonyl]-5-aza-cytidine (N-DEACMOC-dAC) (1a) 7

2.2. 2′-Deoxy-4-*N*-[mono-(2-nitrophenyl)ethoxycarbonyl]-5-azacytidine (N-NPEOC-dAC) (1b) 8

2.2.1. 2-Nitrophenethyl chloroformate (9) 8

2.2.2. 2′-Deoxy-4-*N*-[mono-(2-nitrophenyl)ethoxycarbonyl]-5-azacytidine (N-NPEOC-dAC) (1b)........ 8

2.3. 2′-Deoxy-4-*N*-[mono-[2-(4,5-dimethoxy-2-nitrophenyl)]ethoxycarbonyl]-5-azacytidine (N-DMNPEOC-dAC) (1c) 9

2.3.1. 2-(4,5-Dimethoxy-2-nitrophenyl)ethan-1-ol (11) 9

2.3.2. 2-(4,5-Dimethoxy-2-nitrophenethyl) chloroformate (12) 9

2.3.3. 2′-Deoxy-4-*N*-[mono-[2-(4,5-dimethoxy-2-nitrophenyl)]ethoxycarbonyl]-5-azacytidine (N-DMNPEOC-dAC) (1c) 10

2.4. 4-*N*-[Di-(2-nitrophenyl)ethoxycarbonyl]-5-azacytidine (bis-NPEOC-AC) (1d) 10

2.5. 2′-Deoxy-5′-[mono-7-(diethylamino)-2-oxochroman-4-yl)methoxycarbonyl]-5-aza-cytidine (5′-DEACMOC-dAC) (2) 11

2.5.1. (7-(diethylamino)-2-oxochroman-4-yl)methyl(perfluorophenyl) carbonate (14) 11

2.5.2. 2′-Deoxy-5′-[mono-7-(diethylamino)-2-oxochroman-4-yl)methoxycarbonyl]-5-aza-cytidine (5′-DEACMOC-dAC) (2) 12

2.6. 2′-Deoxy-3′-[mono-7-(diethylamino)-2-oxochroman-4-yl)methoxycarbonyl]-5-aza-cytidine (3′-DEACMOC-dAC) (3) 12

2.6.1. 2′-Deoxy-5′-triisopropyl-5-azacytidine (5′-TIPS-dAC) (15) 12

2.6.2. 2′-Deoxy-3′-[mono-7-(diethylamino)-2-oxochroman-4-yl)methoxycarbonyl]-5′-triisopropyl-5-azacytidine (3′-DEACMOC-5′-TIPS-dAC) (16) 13

2.6.3. 2′-Deoxy-3′-[mono-7-(diethylamino)-2-oxochroman-4-yl)methoxycarbonyl]-5-aza-cytidine (3′-DEACMOC-dAC) (3) 13

3. Supporting data 15

Supporting Figure 1. Molecular model of the catalytic site of deoxycytidine kinase (PDB: 2A30) in complex with 2′-deoxycytidine. 15

Figure S2. UV-Vis absorption spectra of compounds 1a-d 16

Figure S3. Deprotection curves for compounds 1a-d. 17

Figure S4. Mechanism for the photo-induced deprotection of DEACMOC-caged compound. 18

Figure S5. Data on the stability of 3 and proposed mechanisms of dark instability. 19
Figure S6. Control of cellular DNA methylation levels in cells. 20
Figure S7. Compound 3 is not phosphorylated by deoxycytidine kinase (dCK) 21
Figure S8. Western blot analysis on DNMT1 degradation. 22
Figure S9. ^{1}H-NMR spectra of N-DEACMOC-dAC 1a in DMSO 23
Figure S10. ^{13}C-NMR spectra of N-DEACMOC-dAC 1a in DMSO... 24
Figure S11. ^{1}H-NMR spectra of N-NPEOC-dAC 1b in DMSO... 25
Figure S12. ^{13}C-NMR spectra of N-NPEOC-dAC 1b in DMSO 26
Figure S13. ^{1}H-NMR spectra of N-DMNPEOC-dAC 1c in DMSO 27
Figure S14. 13C-NMR spectra of N-DMNPEOC-dAC 1c in DMSO 28
Figure S15. ^{1}H-NMR spectra of bis-NPEOC-AC 1d in DMSO... 29
Figure S16. ^{13}C-NMR spectra of bis-NPEOC-AC 1d in DMSO 30
Figure S17. ^{1}H-NMR spectra of 5′-DEACMOC-dAC 2 in DMSO.. 31
Figure S18. ^{13}C-NMR spectra of 5′-DEACMOC-dAC 2 in DMSO 32
Figure S19. ^{1}H-NMR spectra of 3′-DEACMOC-dAC 3 in DMSO.. 33
Figure S20. ^{13}C-NMR spectra of 3′-DEACMOC-dAC 3 in DMSO 34

1. Supporting methods

All reagents and anhydrous solvents were purchased from Sigma Aldrich or Fischer Scientific unless otherwise stated. 5-azacytidine and 5-aza-2′-deoxycytidine were purchased from Carbosynth Limited. All reactions were performed under argon atmosphere unless otherwise specified. NMR spectra were recorded on 600 MHz on Brucker DRX-600. All chemical shifts (δ) are quoted in ppm relative to residual solvent for ^{1}H-NMR and relative to internal resonance for ^{13}C NMR. Multiplicity is abbreviated as follows: s (singlet), d (doublet), t (triplet) and q (quartet). ESI-MS were recorded on Waters Aquity Ultra performance LC-MS system equipped with an Acquity UPLC BEH C18 column (50 x 2.1 mm, 1.7 μm beads). Reactions were monitored by thin layer chromatography using Merck Millipore TLS Silica gel F254 plates (0.25 mm) which were visualized using UV light at 254 nm. Flash column chromatography was performed with E. Merck silica gel SiO_2 (43-60 μm).

Analysis of photolysis: Into a 2 cm^2 area well of a 24-well tissue culture plate (BD Falcon) were placed 0.5 mL of 100 μM photocaged analogue in DMSO/water (5/95). The solution was irradiated at λ = 365 nm using a Benchtop UV lamp (Model UVGL58, Mineralight lamp, 145 μW cm^{-2}). Aliquots of 20 μL were periodically removed and analysed by HPLC using an Agilent Eclipse C18 column (250 x 4.6 mm, 5 μm) with an elution system of 5-95% acetonitrile in water over 20 min at a flow rate of 1 mL min^{-1}. The percentage of compound converted was determined via integration of HPLC chromatograms.

Testing of the chemical stability: Solutions of **3** or 5′-DEACMOC-dC in DMSO/water (5/95) (1 μM, 1 mL, 1.5 mL Eppendorf® tube) were incubated in the dark at 25 °C using a Thermomixer (Eppendorf® ThermoMixer). Aliquots of 10 μL were periodically removed and analyzed on a Triple Quadrupole 6460 Mass Spectrometer (Agilent Technologies) fitted with an Infinity 1260 LC system (Agilent) and a Hypersil Gold C18 Column (150 x 2.1 mm, 1.9 μm), with an elution system of 5-95% acetonitrile in water with 0.1% formic acid over 20 min at a flow rate of 0.2 mL min^{-1}. Multiple Reaction Monitoring (MRM) was set up and optimised to ensure selective quantitation of the caged-analogue, corresponding photocage and dAC. 2′-deoxyuridine (dU) and 2′-deoxycytidine were used as internal standards.

Testing the effect of the photocage on the activity of dCK kinase: Using the PRECICE® dCK Screening Assay Kit, compound **3**, and controls dAC and 2′-deoxcytidine, (10 μL of 1 mM in DMSO solution) were added to a 96-well plate under subdued light, followed by the addition of 90 μL of standard reaction mix containing 100 mM Tris-HCl, 250 mM KCl, 10 mM $MgCl_2$, 0.5 mg/ml BSA, 5 mM DTT, 5 mM NAD, 1 mM deoxyinosine, 50 mU/ml IMPDH and 5 mU/ml human recombinant dCK. The reaction was started by the addition of ATP solution (10 μL per well) and the increase in absorbance at 340 nm was followed for 30 min at 37 °C. The experiment was repeated by irradiating the compounds to uncage **3** at λ = 365 nm using a benchtop UV lamp (Model 33 UVGL58, Mineralight lamp, 145 μW

cm^{-2}) positioned 20 cm from the well-plate, for 60 min, prior to absorbance measurements.

Cell culture and treatment with compound 3: Osteosarcoma cell line SaOS-2[1] and urinary bladder carcinoma line T24[2] were grown as an adherent monolayer culture in RPMI 1640 medium (Lonza, #12-702F) supplemented with 10% fetal bovine serum (Gibco, #10500064). Cells were seeded at a density of 2 x 10^5 cells into 10 cm cell culture dishes (Cornig, #430176). Accurate cell counts were determined using a Vi-CELL™ XR cell viability analyzer (Beckman Coulter). 72 h after plating, cells were treated with the photocaged analogue **3** or dAC in fresh medium at final concentrations of 0.1, 0.5 and 1.5 μM. Each treatment was administered in triplicates. Following the addition of fresh medium and treatment, one set of plates were subjected to 1 h of UV illumination at λ = 365 nm at 25 °C. The remaining set of plates served as control and were kept in the dark at 25 °C. After 24 h of further incubation at 37 °C cells were collected and genomic DNA was extracted using DNeasy Blood & Tissue Kit (Qiagen, #69504). DNA concentrations were measured using Qubit® dsDNA Assay Kit (Thermo, #Q32854).

Digestion and LC-MS analysis of genomic DNA: 5 μg of genomic DNA was incubated with 5 U of DNA Degradase Plus (Zymo Research) for 4 h at 37 °C. The resulting mixture was spiked with 100 nM of isotope-labelled 2′-deoxycytidine-(^{15}N, D_2) and 5-methyl-2′-deoxycytidine-(D_3) (Toronto Research Chemicals) as internal standards. Synthetic standards 2′-deoxycytidine (C, Sigma), 5-methyl-2′-deoxycytidine (5mC, Berry & Associates) were used to obtain calibration curves in the ranges of 10 – 100 μM for C and 0.5 – 5 μM for 5mdC. 10 μl of nucleic acid digest was injected into an Agilent Infinity 1290 LC system fitted with an Acquity UHPLC HSS T3 column (50 × 2.1 mm, 1.8 μm particle size), maintained at 50 °C, at a flow rate of 300 μL min^{-1}, and a 5 min gradient of 0.1% formic acid in water (buffer A) and 0.1% formic acid in acetonitrile (buffer B) (0-0.5 min 100% A; 0.5-1.4 min 100->70% A; 1.4-2.0 min 70% A; 2.0-3.0 min 70->10% A; 3.0-5.0 min 100% A). The eluent was directed to a Thermo Q Exactive mass spectrometer fitted with a heated electrospray source with temperature set to 350°C. The quantitation was based on the peak area ratio of the analytes to their corresponding isotope-labeled internal standards, and the constructed calibration curves. 5mC levels are expressed as a percentage of total cytosines (C plus 5mC).

Western blot analysis of DNMT1 degradation: Cell pellets containing approx. 10^5 cells were mixed with 5x Laemmli Buffer and incubated at 95°C for 5 min. 30 μg whole cell lysate of each sample was loaded on an 8% SDS PAGE gel and run under standard conditions along with a Precision Plus Protein Standard, 10-250 kD (BioRad, #1610373). Separated proteins were blotted[3] onto a 0.45 μm-pore polyvinylidene (PVDF) membrane (Sigma, #Z671010). The membrane was blocked with 5% Milk/TBST for 1 h at 4°C followed by incubation with primary antibodies (1:200 anti-DNMT1, #sc-271729 and 1:300 anti-GAPDH, #MAB374) overnight at 4°C on a gentle shaking followed by 3 x 10 min rinse in TBST. The membrane was then incubated with the secondary antibodies (1:4000, HRP-

linked Mouse IgG, #NAV931V) for 1 h at RT under gentle shaking, followed by a 5 x 10 min TBST wash. The protein bands were visualized by the addition of 1:1 luminol and hydrogen peroxide mixture (2 mL) (SuperSignalT M West Femto, 34095, Thermo) and incubation in the dark for 10 min. Chemoluminescence was detected using the ODYSSEY CLx Imaging system (LI-COR Biosciences, UK).

2. Procedures for the synthesis of compounds 1a-3

In brief, the synthetic route to **1a** involved modifying NH_2 with the highly reactive chloroformate version of DEACM. The high reactivity helped overcome the amino group's weak nucleophilicity which is a consequence of electron-withdrawal by N5 in the dAC base. Undesired reaction of the 3′- and 5′- OH groups of dAC was suppressed by transient protection with trimethylsilyl[4]. By comparison, **2** was obtained by modifying the sterically highly accessible 5′ OH without any protecting groups for NH_2 or 3′ OH. DEACM was therefore introduced not as chloroformate but as a less reactive pentafluorophenyl ester that solely targets 5′ OH. Similarly, **3** was generated by reacting 3′ OH with the same ester of DEACM. The undesired modification of 5′ OH was blocked by protection with triisopropylsilyl.

2.1. 2′-Deoxy-4-*N*-[mono-7-(diethylamino)-2-oxochroman-4-yl)methoxycarbonyl]-5-aza-cytidine (N-DEACMOC-dAC) (1a)

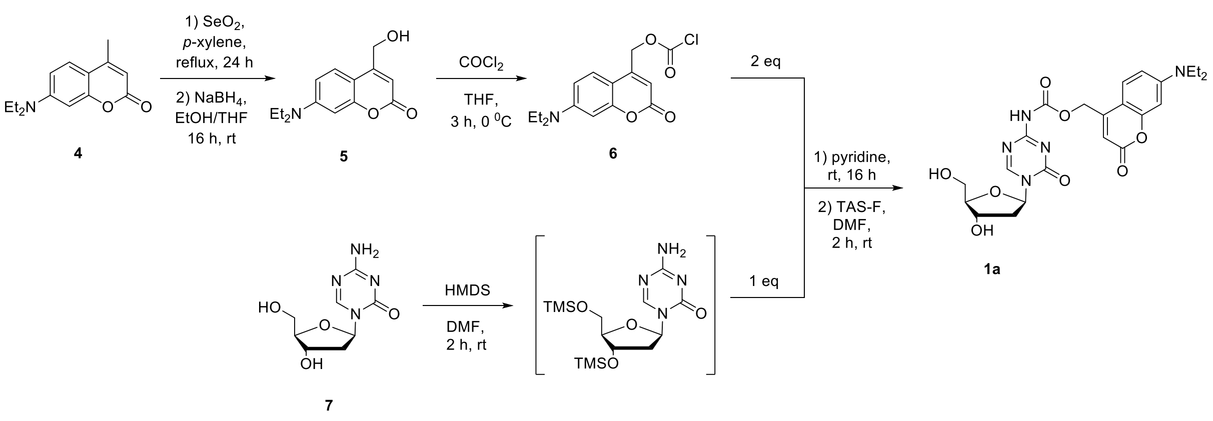

2.1.1. 7-(Diethylamino)-4-(hydroxymethyl)coumarin (5)

Compound **5** was prepared following a published protocol[5] with several adaptations. To a solution of 7-(diethylamino)-4-(methyl)coumarin **4** (4.63 g, 20 mmol, 1 eq) in hot *p*-xylene (120 mL) was added SeO_2 (3.33 g, 30 mmol, 1.5 eq). The mixture was heated under reflux with vigorous stirring overnight. The mixture was filtered and concentrated under reduced pressure. The dark brown residual oil was dissolved in ethanol (130 mL), and sodium borohydride (380 mg, 10 mmol, 0.5 eq) was added to the solution followed by stirring overnight at room temperature. The suspension was carefully hydrolyzed with 1 M HCl (20 mL), diluted with water (30 mL) and extracted with DCM (3 x 50 mL). The organic phase was washed with water (2 x 100 mL), brine (100 mL) and dried over $MgSO_4$ and concentrated *in vacuo*. The crude material was purified by flash chromatography (DCM/acetone 5:1) to yield **5** as a yellow solid (2.71 g, 55%); ^{1}H-NMR (600 MHz, DMSO-d_6) δ 7.39 (d, 1 H, J = 9.0 Hz), 6.62 (dd, 1 H, J = 9.0, 2.7 Hz), 6.48 (d, 1 H, J = 2.7 Hz), 6.03 (s, 1 H), 5.51 (t, 1 H, J = 5.6 Hz), 4.63 (d, 2 H, J = 5.6 Hz), 3.38 (q, 4 H, J = 7.2 Hz), 1.0 (t, 6 H, J = 7 Hz); ^{13}C NMR (600 MHz, DMSO-d_6) δ 161.21, 156.97, 155.64, 150.16, 125.11, 108.53, 105.64, 103.80, 96.73, 59.05, 43.99, 12.17; HRMS (*m/z*) (CI) $[M+H]^+$ calculated for $C_{14}H_{17}NO_3$, 247.1208; found, 247.1203.

2.1.2. 7-(Diethylamino)-4-(hydroxymethyl)coumarin chloroformate (6)

Following an adapted version of a published procedure[6], a solution of **5** (250 mg, 1 mmol, 1 eq) in dry THF (18 mL) was treated with *N,N*-diisopropylethylamine (260 µL, 1.5 mmol, 1.5 eq) and stirred for 5 minutes at 0 ^{0}C. After dropwise addition of phosgene in toluene 20% w/w (1.25 mL, 2.5 mmol, 2.5 eq), stirring was continued in the dark at 0 ^{0}C for 3 h. The reaction mixture was transferred onto a mixture of ethyl acetate and water (1:1, 200 mL) and the layers were separated and the organic layer was dried over $MgSO_4$ and concentrated *in vacuo*. LC-MS analysis indicated about 70% conversion of starting material to the chloroformate. Due to its high reactivity, the crude chloroformate **6** was used in the next step without further purification.

2.1.3. 2′-Deoxy-4-*N*-[mono-7-(diethylamino)-2-oxochroman-4-yl)methoxy carbonyl]-5-aza-cytidine (N-DEACMOC-dAC) (1a)

5-aza-2′-deoxycytidine, **7** (228 mg, 1 mmol, 1 eq) was co-evaporated with anhydrous pyridine (3 x 5 mL) and subsequently suspended into anhydrous DMF (10 mL) and treated with hexamethyldisilazane (0.53 mL, 2.5 mmol, 2.5 eq), using a modified procedure[4]. After 2 h of stirring at ambient temperature, the solution was concentrated and dried by evaporation from toluene (3 x 5 mL), followed by anhydrous pyridine (3 x 5 mL). The resultant residue was suspended in anhydrous pyridine (20 mL) to which chloroformate **6** (2 mmol, 2 eq) in dry DCM (8 mL) was added and the mixture was left stirring in the dark overnight. The solution was concentrated to dryness and the resultant residue was co-evaporated with anhydrous toluene (3 x 5 mL) and suspended into anhydrous DMF (10 mL). Silyl deprotection was achieved by the addition of TAS-F (600 mg, 2.17 mmol, 3.57 eq). After 2 h, the mixture was concentrated *in vacuo* and co-evaporated with anhydrous toluene (3 x 5 mL). The crude material was purified by flash chromatography (0-10% MeOH/DCM) to yield the title compound as a yellow solid (46 mg, 14%); 5% MeOH/DCM $R_f = 0.45$). ^{1}H-NMR (600 MHz, DMSO-*d*$_6$) δ 11.04 (br, 1 H), 8.86 (s, 1 H), 7.47 (d, 1 H, *J* = 9.0 Hz), 6.70 (dd, 1 H, *J* = 9.2, 2.4 Hz), 6.55 (d, 1 H, *J* = 2.4 Hz*)*, 6.17 (s, 1 H), 6.00 (t, 1 H, *J* = 6.3 Hz*)*, 5.37 (s, 2 H), 5.29 (d, 1 H, *J* = 4.4 Hz), 5.14 (t, 1 H, *J* = 5.2 Hz), 4.25 (m, 1 H), 3.87 (q, 1 H, *J* = 3.8 Hz), 3.65 (ddd, 1 H, *J* = 11.7, 5.2, 3.6 Hz), 3.57 (ddd, 1 H, *J* = 11.7, 5.2, 3.6 Hz), 3.43 (q, 4 H, *J* = 7.2 Hz), 2.31 (ddd, 1 H, *J* = 13.6, 6.5, 4.7 Hz), 2.22 (dt, 1 H, *J* = 13.4, 5.9 Hz), 1.12 (t, 6 H, *J* = 7.0 Hz); ^{13}C NMR (600 MHz, DMSO-*d*$_6$) δ 160.78, 160.73, 157.56, 155.76, 153.04, 150.76, 150.48, 125.45, 108.77, 105.07, 104.88, 96.86, 88.13, 86.43, 69.32, 61.99, 60.45, 44.05, 40.68, 12.32. HRMS (*m/z*) (ESI) $[M+Na]^+$ calculated for $C_{23}H_{27}N_5O_8Na$, 524.1757; found, 524.1755.

2.2. 2′-Deoxy-4-*N*-[mono-(2-nitrophenyl)ethoxycarbonyl]-5-azacytidine (N-NPEOC-dAC) (1b)

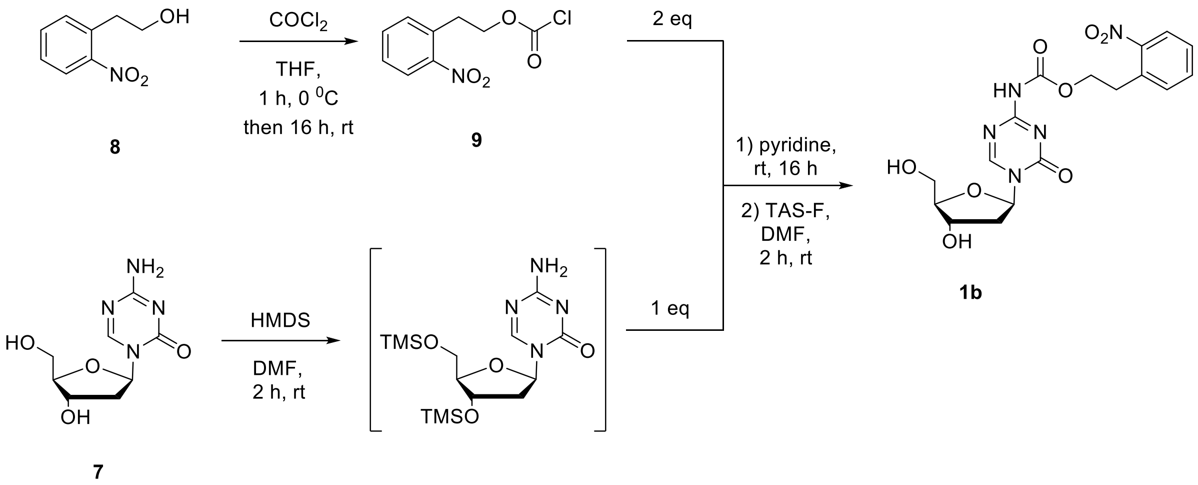

2.2.1. 2-Nitrophenethyl chloroformate (9)

A solution of phosgene in toluene (7.50 mL, 20% w/w, 14.18 mmol, 2 eq) was added to a suspension of 2-nitrophenethyl alcohol, **8** (1.00 mL, 7.11 mmol, 1 eq) in THF (20 mL) at 0 ^{0}C[7]. The suspension was stirred for 1 h, then at room temperature overnight. TLC analysis (5% MeOH/DCM) suggested the complete conversion of the starting material (R_f = 0.48) to the chloroformate (R_f = 0.80). The excess phosgene was removed under reduced pressure and quenched with aqueous 0.1 M NaOH. Compound **9** was isolated (1.53 g, 94%) as a yellow oil. ^{1}H-NMR (600 MHz, $CDCl_3$) δ 7.96 (dd, 1 H, *J* = 1.0, 7.6 Hz), 7.58 (td, 1 H, *J* = 1.0, 7.6 Hz), 7.43 (td, 1 H, *J* = 1.2, 7.6 Hz), 7.39 (dd, 1 H, *J* = 1.2, 7.6 Hz), 4.61 (t, 2 H, *J* = 6.5 Hz), 3.31 (t, 2 H, *J* = 6.5 Hz); ^{13}C NMR (600 MHz, $CDCl_3$) δ 150.55, 149.33, 133.63, 133.08, 131.67, 128.60, 125.32, 71.19, 32.40; HRMS (*m/z*) $[M+H]^+$ calculated for $C_9H_8ClNO_4$, 229.0142; found, 229.0033.

2.2.2. 2′-Deoxy-4-*N*-[mono-(2-nitrophenyl)ethoxycarbonyl]-5-azacytidine (N-NPEOC-dAC) (1b)

Synthesized from 2-nitrophenethyl chloroformate **9** and 5-aza-2′-deoxycytidine **7,** following the procedure described in 2.1.3**.** The title compound was isolated as a white foam (231 mg, 53%); 5% MeOH/DCM R_f = 0.16; ^{1}H-NMR (600 MHz, DMSO-*d₆*) δ 10.71 (br, 1 H), 8.81 (s, 1 H), 7.97 (dd, 1 H, *J* = 8.1, 1.1 Hz), 7.67 (td, 1 H, *J* = 7.6, 1.2 Hz), 7.60 (dd, 1 H, *J* = 7.7, 1.1 Hz), 7.54-7.50 (m, 1 H), 5.98 (t, 1 H, *J* = 5.1 Hz), 5.36 (d, 1 H, *J* = 4.4 Hz), 5.19 (t, 1 H, *J* = 5.1 Hz), 4.36 (t, 2 H, *J* = 6.5 Hz), 4.25-4.22 (m, 1 H), 3.87 (q, 1 H, *J* = 3.7 Hz), 3.64 (ddd, 1 H, *J* = 12.0, 5.0, 3.5Hz), 3.57 (ddd, 1 H, *J* = 12.0, 5.0, 3.5 Hz), 3.19-3.15 (m, 2 H), 2.30 (ddd, 1 H, *J* = 13.3, 6.3, 4.8 Hz), 2.20 (dt, 1 H, *J* = 13.4, 5.9 Hz); ^{13}C NMR (600 MHz, DMSO-d_6) δ 162.46, 157.33, 153.07, 150.73, 149.32, 133.42, 132.95, 132.13, 128.25, 124.53, 88.02, 86.36, 69.31, 64.38, 60.42, 40.63, 31.41; HRMS (*m/z*) $(ESI)^-$ $[M]^-$ calculated for $C_{17}H_{19}N_5O_8$, 420.1155; found. 420.1135.

2.3. 2′-Deoxy-4-*N*-[mono-[2-(4,5-dimethoxy-2-nitrophenyl)]ethoxycarbonyl]-5-azacytidine (N-DMNPEOC-dAC) (1c)

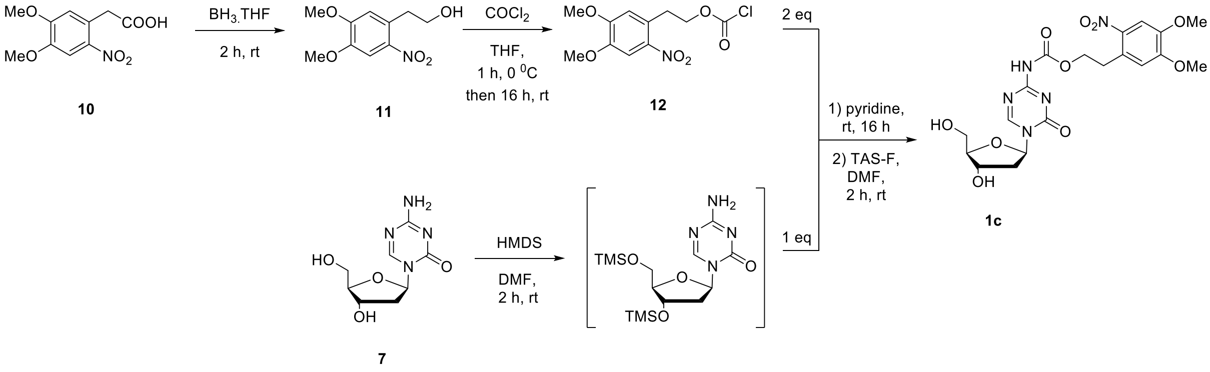

2.3.1. 2-(4,5-Dimethoxy-2-nitrophenyl)ethan-1-ol (11)

Following a published procedure[8], a solution of 2-(4,5-dimethoxy-2-nitrophenyl)acetic acid **10** (519 mg, 2.15 mmol, 1 eq) in dry THF (13 mL) was mixed with BH_3·THF (1.0 M, 5 mL, 5 mmol, 2.3 eq) under argon atmosphere. The solution was allowed to stir at room temperature for 2 h, then H_2O (2 mL) was slowly added to quench the reaction. The crude mixture was concentrated *in vacuo* and the resultant residue was diluted in ethyl acetate (100 mL) and washed with saturated $NaHCO_3$ (2 x 60 mL) and brine (60 mL), dried over $MgSO_4$ and concentrated *in vacuo* to afford **11** (476 mg, 98%) as a yellow precipitate: R_f = 0.62 (5% MeOH/DCM); ^{1}H-NMR (600 MHz, DMSO-d_6) δ 7.54 (s, 1 H), 7.03 (s, 1 H), 4,74 (t, 1 H, *J* = 5.3 Hz*)*, 3.87 (s, 3 H), 3.82 (s, 3 H), 3.62 (q, 2 H, *J* = 6.8 Hz), 3.02 (t, 2 H, *J* = 6.8 Hz); ^{13}C NMR (600 MHz, DMSO-d_6) δ 152.49, 146.89, 141.32, 128.86, 114.52, 107.88, 61.11, 56.13, 55.95, 36.01; HRMS (*m/z*) (CI) $[M+H]^+$ calculated for $C_{10}H_{13}NO_5$, 227.0788; found, 227.0789.

2.3.2. 2-(4,5-Dimethoxy-2-nitrophenethyl) chloroformate (12)

A solution of phosgene in toluene (2.50 mL, 20% w/w, 4.73 mmol, 2.4 eq) was added to a suspension of **11** (450 mg, 1.98 mmol, 1 eq) in THF (10 mL) at 0 ^{0}C. The suspension was stirred at for 1 h, then at room temperature overnight. The excess phosgene was removed under reduced pressure and quenched with aqueous 0.1 M NaOH. The title compound **12** was isolated (550 mg, 96%) as a dark yellow precipitate: R_f = 0.94 (5% MeOH/DCM); ^{1}H-NMR (600 MHz, $CDCl_3$) δ 7.66 (s, 1 H), 6.73 (s, 1 H), 4.65 (t, 2 H, *J* =6.3 Hz), 3.98 (s, 3 H), 3.95 (s, 3 H), 3.35 (t, 2 H, *J* = 6.3 Hz); ^{13}C NMR (600 MHz, $CDCl_3$) δ 153.25, 150.60, 148.20, 141.34, 126.74, 114.32, 108.52, 71.36, 56.62, 56.49, 33.22; HRMS (*m/z*) (CI) $[M+H]^+$ calculated for $C_{11}H_{13}ClNO_6$, 290.0426; found, 290.0419.

2.3.3. 2′-Deoxy-4-*N*-[mono-[2-(4,5-dimethoxy-2-nitrophenyl)]ethoxycarbonyl]-5-azacytidine (N-DMNPEOC-dAC) (1c)

Synthesized from chloroformate **12** and 5-aza-2′-deoxycytidine **7,** following the procedure described in 2.1.3**.** The title compound was isolated as a solid yellow foam (140 mg, 28%); 5% MeOH/DCM R_f = 0.39; ^{1}H-NMR (600 MHz, DMSO-d_6) δ 10.73 (br, 1 H), 8.82 (s, 1 H), 7.61 (s, 1 H), 7.05 (s, 1 H), 5.99 (t, 1 H, *J* = 6.0 Hz), 5.28 (d, 1 H, *J* = 4.4 Hz), 5.13 (t, 1 H, *J* = 5.1 Hz), 4.37 (t, 2 H, *J* = 6.5 Hz), 4.25-4.22 (m, 1 H), 3.89 (s, 3 H), 3.88-3.87 (m, 1 H), 3.84 (s, 3 H), 3.67-3.63 (m, 1 H), 3.59-3.53 (m, 1 H), 3.20 (t, 2 H, *J* = 6.5 Hz), 2.30 (ddd, 1 H, *J* = 13.3, 6.3, 4.8 Hz), 2.21 (dt, 1 H, *J* = 13.4, 5.9 Hz); ^{13}C NMR (600 MHz, DMSO-d_6) δ 163.46, 157.35, 152.74, 150.69, 147.28, 141.00, 127.37, 114.75, 108.06, 88.07, 86.33, 69.32, 64.38, 60.44, 56.25, 55.97, 40.64, 32.20; HRMS (*m/z*) (ESI) $[M+Na]^+$ calculated for $C_{19}H_{22}N_5O_{10}Na$, 480.1367; found, 480.1369.

2.4. 4-*N*-[Di-(2-nitrophenyl)ethoxycarbonyl]-5-azacytidine (bis-NPEOC-AC) (1d)

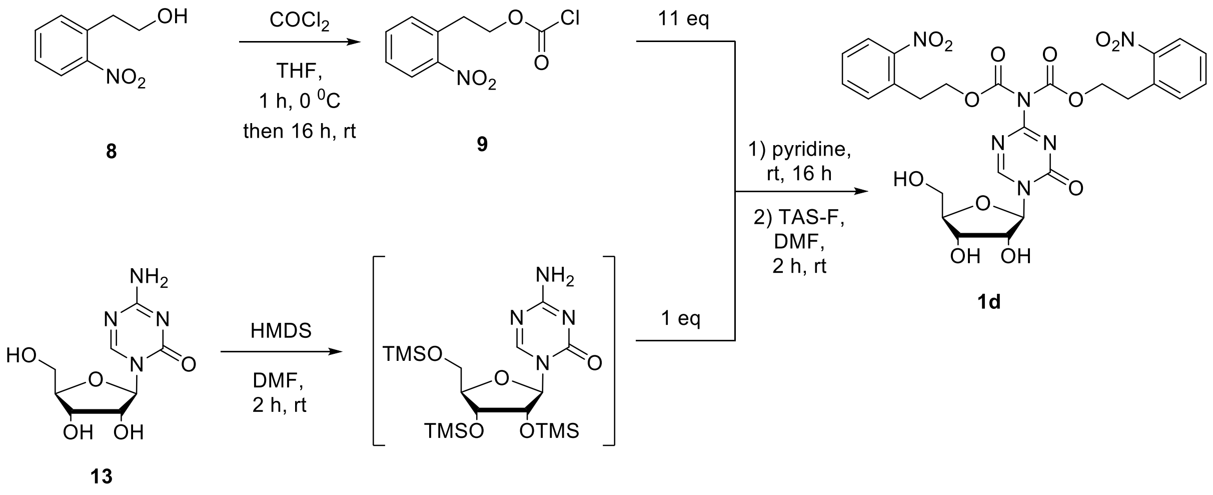

5-Azacytidine **13** (150 mg, 0.61 mmol, 1 eq) was co-evaporated with anhydrous pyridine (3 x 5 mL) and subsequently suspended into anhydrous DMF (10 mL) before adding hexamethyldisilazane (650 µl, 3.11 mmol, 5.11 eq). After stirring for 2 hours at ambient temperature, the solution was concentrated and the residue dried by three cycles of evaporation from toluene (5 mL), followed by anhydrous pyridine (3 x 5 mL). The resultant residue was subsequently suspended into anhydrous pyridine (30 mL) to which 2-nitrophenethyl chloroformate **9** (see 2.2.1) (1.53 g, 6.69 mmol, 11 eq) in anhydrous DCM (8 mL) was added and the resultant mixture was stirred at room temperature overnight. The solvent was removed under reduced pressure and the resultant residue was co-evaporated with toluene (3 x 10 mL) and suspended in anhydrous DMF (10 mL). Silyl deprotection was achieved with the

addition of TAS-F (600 mg, 2.17 mmol, 3.57 eq). After 2 h, the mixture was concentrated *in vacuo* and the resultant residue was co-evaporated with anhydrous toluene (3 x 5 mL). The crude material was purified by flash chromatography (0-10% EtOH/DCM) to yield **1d** as a colorless film (56.9 mg, 14%); R_f = 0.58; ^{1}H-NMR (600 MHz, DMSO-d_6) δ 9.10 (s, 1 H), 7.93 (d, 2 H, *J* = 8.2 Hz), 7.64 (td, 2 H, *J* = 7.6, 1.2 Hz), 7.51-7.48 (m, 4 H), 5.74 (d, 1 H, *J* = 4.8 Hz), 5.68 (d, 1 H, *J* = 1.4 Hz), 5.34 (t, 1 H, *J* = 4.6 Hz), 5.11 (d, 1 H, *J* = 6.8 Hz), 4.46 (m, 4 H), 4.14 (dt, 1 H, *J* = 4.7, 2.4 Hz), 4.09 (dt, 1 H, *J* = 7.0, 3.5 Hz), 3.97 (dt, 1 H, *J* = 7.8, 2.2 Hz), 3.85 (ddd, 1 H, *J* = 12.0, 4.5, 2.6 Hz), 3.64 (ddd, *J* = 12.1, 4.4, 2.1 Hz), 3.18 (t, 4 H, *J* = 6.2 Hz); ^{13}C NMR (600 MHz, DMSO-d_6) δ 163.59, 159.03 , 152.66, 150.16, 149.31, 133.36, 132.67, 131.78, 128.134, 118.14, 91.24, 83.97, 74.02, 67.66, 67.50 , 58.86, 30.62, HRMS (*m/z*) (ESI) $[M+Na]^+$ calculated for $C_{26}H_{26}N_6O_{13}Na$, 653.1456; found, 653.1456.

2.5. 2′-Deoxy-5′-[mono-7-(diethylamino)-2-oxochroman-4-yl)methoxycarbonyl]-5-aza-cytidine (5′-DEACMOC-dAC) (2)

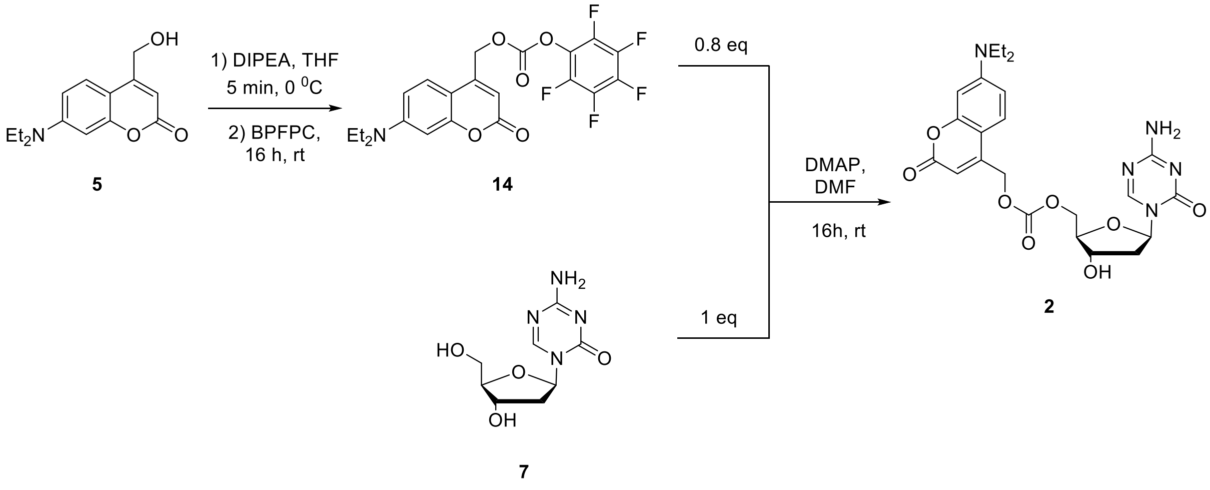

2.5.1. (7-(diethylamino)-2-oxochroman-4-yl)methyl(perfluorophenyl) carbonate (14)

A solution of **5** (300 mg, 1.2 mmol, 1 eq) in dry THF (10 mL) was treated with *N,N*-Diisopropylethylamine (261 µL, 1.5 mmol, 1.3 eq) and stirred for 5 minutes at 0 °C. Bis(pentafluorophenyl)carbonate (956 mg, 2.4 mmol, 2 eq) was then added and stirring was continued at room temperature under the exclusion of light overnight. The reaction mixture was transferred onto a mixture of ethyl acetate and water (1:1, 200 mL) and the layers were separated and the organic layer was dried over MgSO4 and concentrated *in vacuo*. The crude was purified via flash column chromatography (neat DCM) to obtain **14** as a yellow oil (518 mg, 94%); ^{1}H-NMR (600 MHz, DMSO-*d6*) δ 7.29 (d, 1 H, *J* = 9.0 Hz), 6.61 (dd, 1 H, *J* = 9.0, 2.6 Hz), 6.53(d, 1 H, *J* = 2.7 Hz), 6.22 (t, 1 H, *J* = 1.1 Hz), 5.44 (d, 1 H, *J* = 1.1 Hz), 3.43 (q, 4 H, *J* = 7.2 Hz), 1.22 (t, 6 H, *J* = 7.1 Hz); 13C NMR

(600 MHz, DMSO-*d6*) δ 161.97, 156.45, 151.18, 151.05, 147.41, 124.35, 109.02, 106.95, 105.53, 98.00 (C-9), 66.97, 44.93, 12.52; LCMS (ESI+) [M] *m/z* calculated for $C_{21}H_{16}F_5NO_5$ 457.35302, found 457.11

2.5.2. 2′-Deoxy-5′-[mono-7-(diethylamino)-2-oxochroman-4-yl)methoxycarbonyl]-5-aza-cytidine (5′-DEACMOC-dAC) (2)

5-aza-2'-deoxycytidine **7** (157 mg, 0.69 mmol, 1 eq), **14** (518 mg, 1.13 mmol, 1.6 eq) and DMAP (4 mg, 0.03 mmol, 0.04 eq) were dissolved in anhydrous DMF (6 mL) and stirred at room temperature under inert atmosphere for 16 h. The reaction mixture was concentrated and coevaporated with anhydrous toluene (3 x 3 mL). The residue was dissolved in DCM (50 mL) and washed with water. The organic layer was dried and concentrated *in vacuo*. The crude was dry-loaded onto and purified *via* flash column chromatography (0-10% MeOH/DCM) and recrystallized in methanol to yield **2** (76 mg, 22 %) as a yellow solid. ^{1}H-NMR (600 MHz, DMSO-*d6*) δ 8.30 (s, 1 H), 7.56 (d, 2 H, *J* = 33.0 Hz), 7.46 (dd, 1 H, *J* = 9.0 Hz), 6.70 (dd, *J* = 9.1, 2.6 Hz), 6.55 (d, 1 H, *J* = 2.5 Hz), 6.04 (t, 1 H, *J* = 6.4 Hz), 6.02 (s, 1 H), 5.46 (d, 1 H, *J* = 4.5 Hz), 5.36 (s, 2 H), 4.39 (dd, 1 H, *J* = 11.6, 3.7 Hz), 4.34 (dd, 1 H, *J* = 11.6, 6.4 Hz), 4.27 (dq, 1 H, *J* = 6.5, 4.4 Hz), 4.00 (ddd, 1 H, *J*=6.4, 4.2, 3.9 Hz), 3.43 (q, 4 H, *J* = 7.1), 2.30 (ddd, 1 H, *J* = 13.6, 6.7, 6.3 Hz), 2.21 (ddd, 1 H, *J*=13.6, 6.7, 4.6 Hz), 1.12 (t, 6 H, *J* = 7.1); ^{13}C NMR (600 MHz, DMSO-*d6*) δ 166.07, 160.66, 156.13, 156.06, 153.97, 153.29, 152.74, 150.34, 125.60, 108.61, 106.78, 104.92, 96.98, 85.53, 83.68, 70.02, 66.88, 64.67, 43.60, 40.48, 12.04. *m/z* (ESI) [M+H]+ calculated for $C_{23}H_{28}N_5O_8$ 502.1938, found 502.1917

2.6. 2′-Deoxy-3′-[mono-7-(diethylamino)-2-oxochroman-4-yl)methoxycarbonyl]-5-aza-cytidine (3′-DEACMOC-dAC) (3)

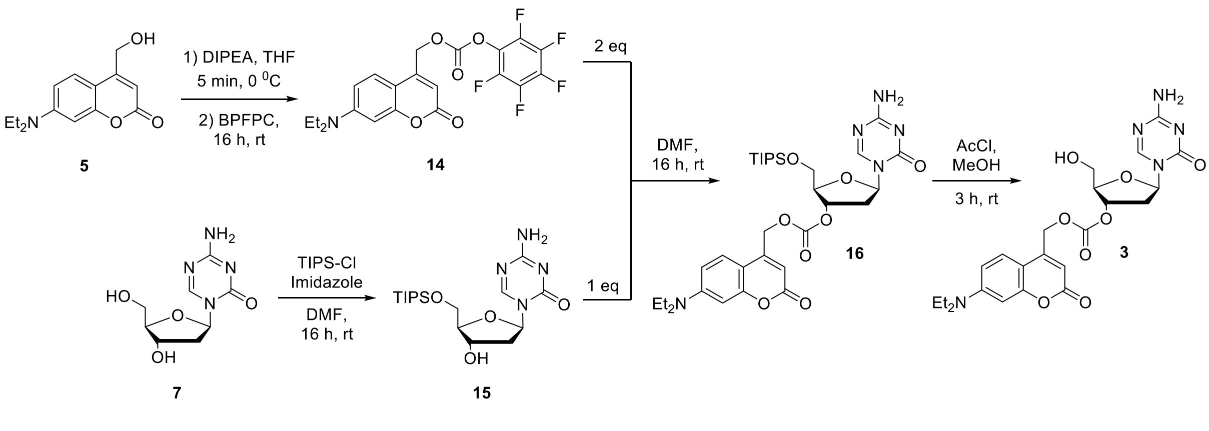

2.6.1. 2′-Deoxy-5′-triisopropyl-5-azacytidine (5′-TIPS-dAC) (15)

To a stirred, cooled solution of 5-aza-2'-deoxycytidine **7** (150 mg, 0.66 mmol, 1 eq), imidazole (98 mg, 1.45 mmol, 2.2 eq) and DMAP (8 mg, 0.07 mmol, 0.1 eq) in anhydrous DMF (4 mL) was added a solution of triisopropyl silyl chloride (154 μL, 0.72 mmol, 1.1 eq) in anhydrous DMF (2 mL). The

mixture was stirred for 1 h at 0 ^{0}C and overnight at room temperature. The crude was concentrated and purified *via* flash column chromatography (0-5% MeOH/DCM) to obtain the title compound as a white solid (133 mg, 52.4%). ^{1}H-NMR (600 MHz, DMSO-*d6*) δ 8.38 (s, 1 H), 7.51 (d, 2 H, *J* = 14.0 Hz), 6.04 (t, 1 H, *J* = 6.4 Hz), 5.28 (d, 1 H, *J* = 4.4 Hz), 4.27 (m, 1 H), 3.91-3.87 (m, 1 H), 3.88-3.86 (m, 1 H), 3.83-3.80 (m, 1 H), 2.26 (ddd, 1 H, *J* = 13.5, 6.6, 4.1 Hz), 2.21 (ddd, 1 H, *J* = 13.6, 6.4, 6.4 Hz), 1.14-1.08 (m, 3 H), 1.04 (d, 18 H, *J* = 7.0 Hz) ; ^{13}C NMR (600 MHz, DMSO-***d6***) δ 165.96, 155.38, 153.03, 84.30, 85.14, 69.78, 63.10, 40.63, 17.67, 11.34. *m/z* (ESI) [M+H]+ calculated for $C_{17}H_{32}N_4O_4Si$ 384.21928, found 384.55200.

2.6.2. 2′-Deoxy-3′-[mono-7-(diethylamino)-2-oxochroman-4-yl)methoxycarbonyl]-5′-triisopropyl-5-azacytidine (3′-DEACMOC-5′-TIPS-dAC) (16)

To a stirred solution of **15** (364 mg, 0.94 mmol, 1 eq) and DMAP (11 mg, 0.09 mmol, 0.1 eq) in anhydrous DMF (4 mL) was added a solution of **14** (340 mg, 0.74 mmol, 0.8 eq) in anhydrous DMF (4 mL) at 0 ^{0}C. The mixture was stirred for 6 h and was concentrated *in vacuo*. Traces of DMF were removed via coevaporation with toluene (3 x 3 mL). The crude was purified *via* flash column chromatography (0-5% MeOH/DCM) to obtain **16** as a yellow solid (191 mg, 30.9%). ^{1}H-NMR (600 MHz, DMSO-*d6*) δ 8.35 (s, 1 H), 7.62 (d, 2 H, *J* = 20.1 Hz), 7.48 (d, 1 H, *J* = 9.1 Hz), 6.71 (dd, *J* = 9.2, 2.6 Hz), 6.56 (d, 1 H, *J* = 2.5 Hz), 6.05 (dd, 1 H, *J* = 7.8, 6.2 Hz), 6.02 (s, 1 H), 5.36 (s, 2 H), 5.24 (dt, 1 H, *J* = 6.5, 2.3 Hz), 4.25 (q, 1 H, *J* = 3.0 Hz), 3.92 (dd, 1 H, *J* = 11.1, 3.75 Hz), 3.87 (dd, 1 H, *J* = 11.2, 3.9 Hz), 3.43 (dt, 4 H, *J* = 7.6, 6.8 Hz), 2.58 (ddd, 1 H, *J* = 14.3, 5.9, 2.4 Hz), 2.40 (ddd, 1 H, *J* = 14.5, 7.6, 6.5 Hz), 1.12 (t, 6 H, *J* = 7.0 Hz), 1.12-1.05 (m, 3 H), 1.01 (d, 18 H, *J* = 6.9 Hz); ^{13}C NMR (600 MHz, DMSO-*d6*) δ 165.90, 160.56, 155.93, 155.43, 153.36, 153.36, 152.86, 150.55, 125.72, 108.82, 105.84, 105.21, 96.87, 85.48, 84.60, 65.06, 63.83, 63.39, 44.04, 39.38, 17.61, 12.34, 11.26, *m/z* (ESI) [M+H]+ calculated for $C_{32}H_{47}N_5O_8Si$ 658.3267, found 658.3268.

2.6.3. 2′-Deoxy-3′-[mono-7-(diethylamino)-2-oxochroman-4-yl)methoxycarbonyl]-5-aza-cytidine (3′-DEACMOC-dAC) (3)

To a stirred solution of **16** (87 mg, 0.13 mmol, 1 eq) in dry methanol (3 mL), acetyl chloride (20 µL, 0.28 mmol, 2 eq) was added under inert atmosphere. The mixture was stirred at room temperature for 3 h in the dark. It was then partially concentrated and diluted with DCM (20 mL) and neutralized with 10% sodium bicarbonate solution. The organic layer was washed with water (10 mL), dried and concentrated. The crude was purified *via* flash column chromatography (0-8% MeOH/DCM) to obtain **3** (36 mg, 56%) as a yellow solid. ^{1}H-NMR (600 MHz, DMSO-*d6*) δ 8.47 (s, 1 H), 7.59 (dd, 2 H, *J* = 13.7, 2.3 Hz), 6.72 (dd, 1 H, *J* = 9.1, 2.6 Hz), 6.56 (dd, *J* = 9.2, 2.6 Hz), 6.56 (d, 1 H, *J* = 2.5 Hz), 6.05 (dd, 1 H, *J* = 8.1, 6.0 Hz), 6.02 (s, 1 H), 5.36 (s, 2 H), 5.22 (t, 1 H, *J* = 5.3 Hz), 4.17 (q, 1 H, *J* = 3.8, 2.0 Hz), 3.64 (m, 2 H), 3.43 (q, 4 H, *J* = 7.1 Hz), 2.43 (ddd, 1 H, *J* = 14.3, 8.3, 6.1 Hz), 1.12 (t, 6 H, *J*

= 7.1 Hz); ^{13}C NMR (600 MHz, DMSO-*d6*) δ 165.66, 160.59, 156.02, 155.90, 153.44, 153.03, 150.56, 149.68, 125.66, 106.66, 105.66, 105.18, 96.88, 85.40, 84.94, 79.26, 64.95, 61.25, 44.05, 37.53, 12.34, *m/z* (ESI) [M+H]+ calculated for $C_{23}H_{27}N_5O_8$ 502.1932, found 502.1929.

3. Supporting data

Supporting Figure 1. Molecular model of the catalytic site of deoxycytidine kinase (PDB: 2A30) in complex with 2′-deoxycytidine. (a) Surface analysis of the binding pocket of 2′-deoxycytidine kinase illustrating that 5-aza-2′-deoxycytidine modified at N4 would not fit into the catalytic site. There is a tight interaction between the exocyclic N4 and the amino acid residues Asp133 and Gln197 lining the pocket, as indicated by the white arrow. (b) Chemical diagram illustrating the interaction of 5-aza-2′-deoxycytidine with amino acid side chains of the kinase. The model and the diagram were generated with CPP4 and PoseView software.

a

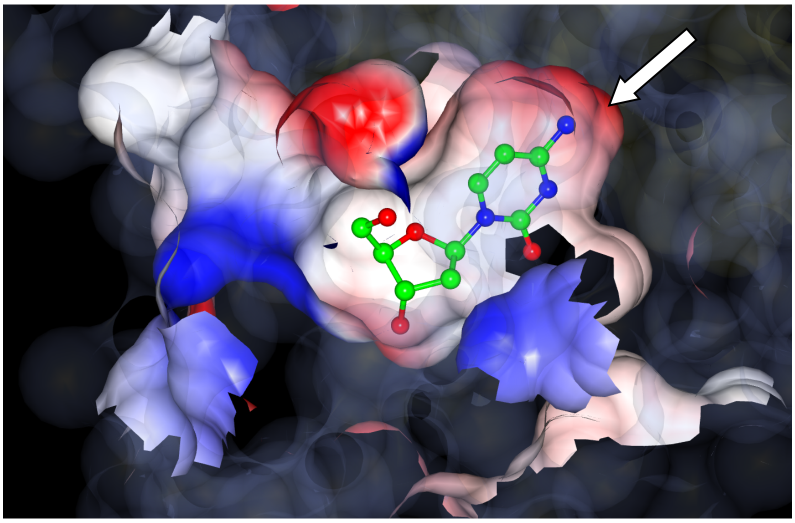

b

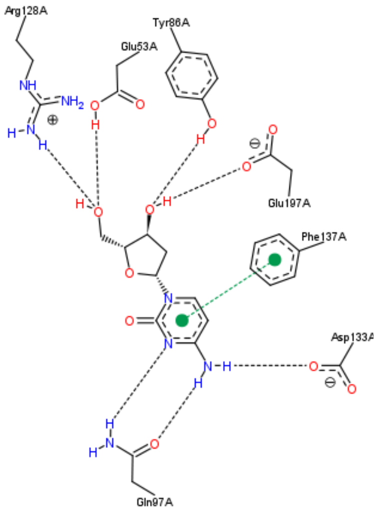

Figure S2. UV-Vis absorption spectra of compounds 1a-d. N-DEACMOC-dAC (1a) N-NPEOC-dAC (1b), N-DMNPEOC-dAC (1c), and bis-NPEOC-AC (1d).

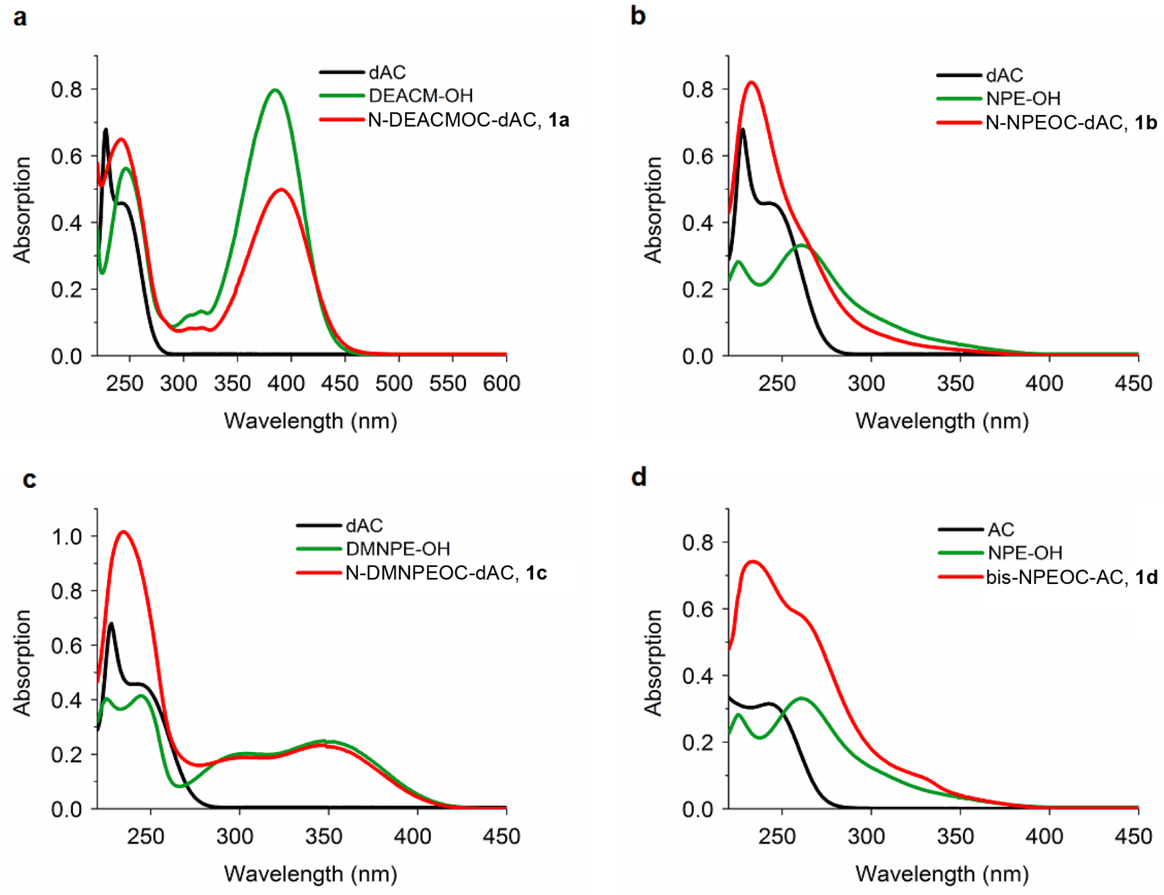

Figure S3. Deprotection curves for compounds 1a-d. The graphs plot the change in molar ratio of caged compounds **1a-d** and photo-deprotected dAC (a-c) and AC (d) over time upon irradiation at λ = 365 nm (a, c) or λ = 254 nm (b, d). 1% acetic acid was included in the photolysis of compound **1d** in order to reduce hydrolytic instability.

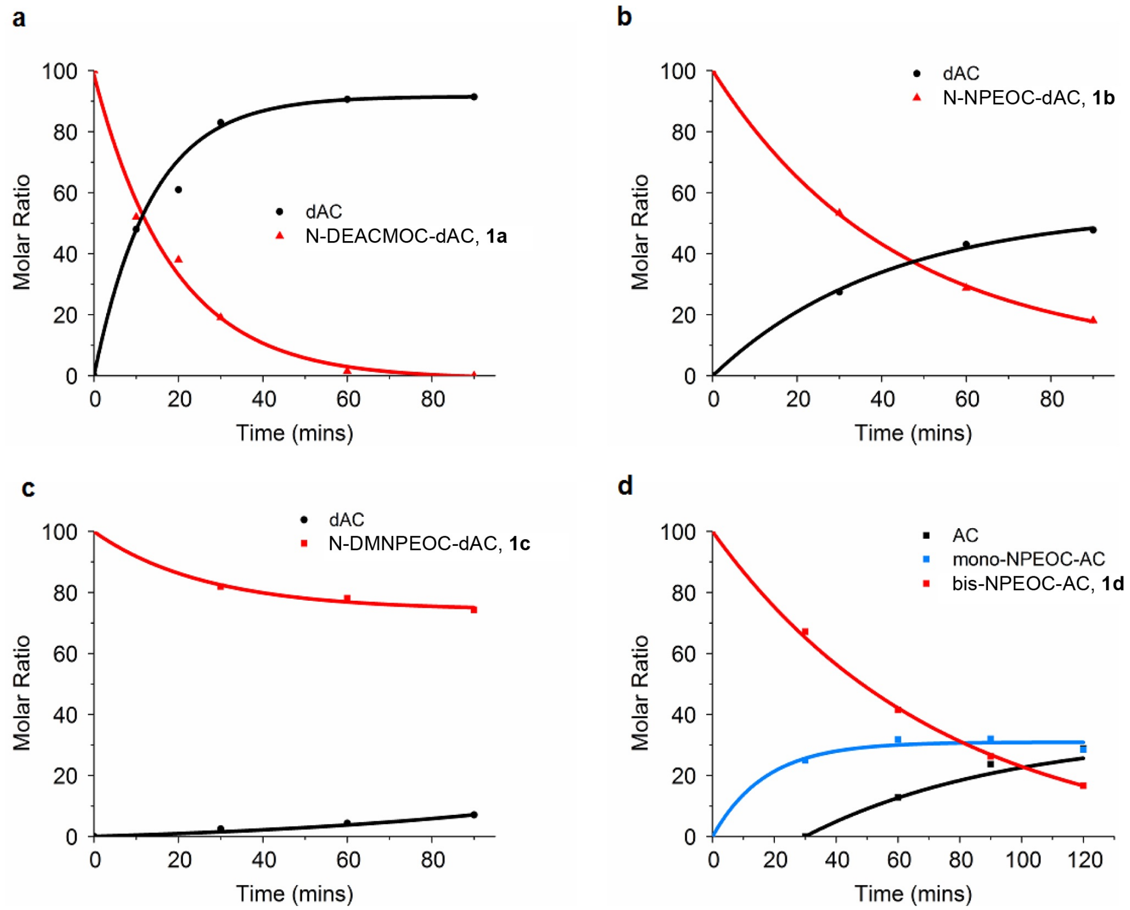

Figure S4. Mechanism for the photo-induced deprotection of DEACMOC-caged compound. Upon initial photo-absorption, relaxation to the lowest (π,π*) excited singlet state is followed by heterolytic C-O bond cleavage. The resulting coumarin-cation reacts with solvent to form a stable product while the liberated carboxylic acid undergoes decarboxylation to yield free alcohol.

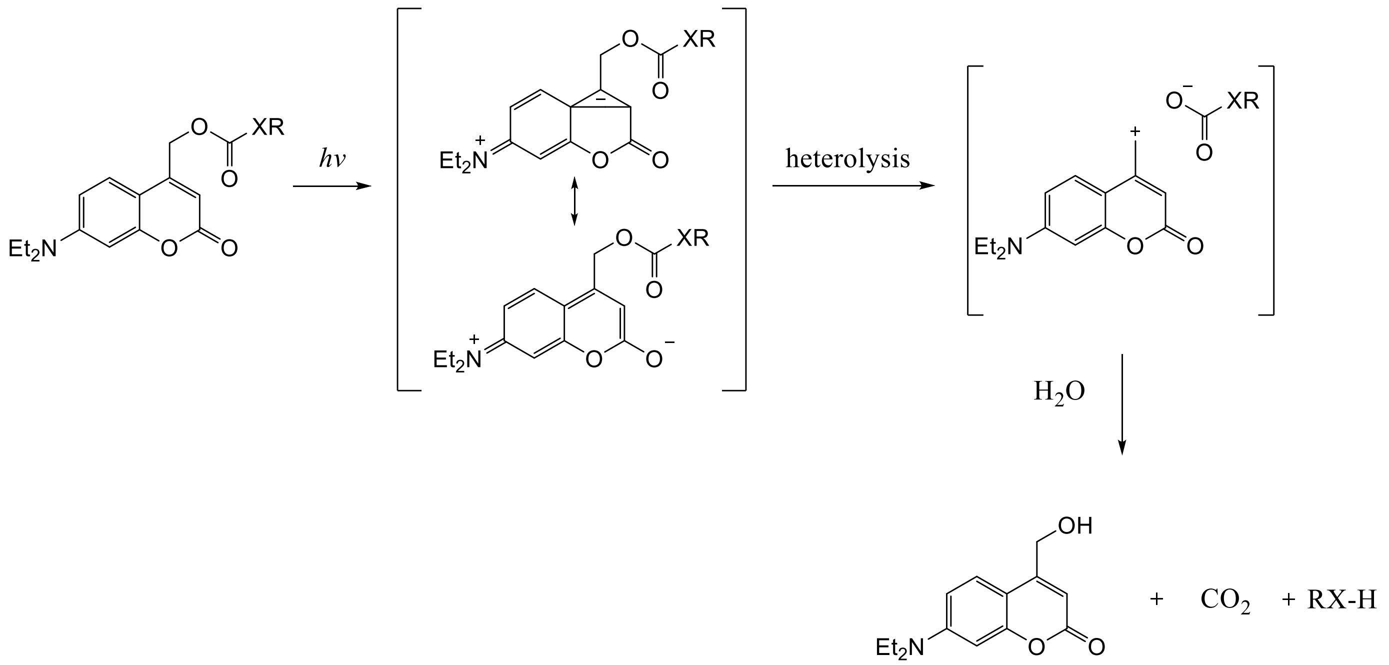

Figure S5. Data on the stability of 3 and proposed mechanisms of dark instability. (A) Stability traces of compound **3**, 3'-DEACMOC-deoxycytidine (3'CdC), and dAC in 10 mM HEPES, pH 7.2 at 25°C. The instability of 3'C-dC represents the carbonate link instability, while 3 includes both carbonate and heterocycle instability, and dAC the heterocycle instability. The stability traces were determined with HPLC (dAC) and with LC-MS/MS (**3**, 3'-DEACMOC-deoxycytidine). (B) Photocaged analogue **3** may undergo hydrolytic ring opening at the triazine ring (blue) or/and hydrolytic cleavage of the carbonate linkage. The triazine ring opening is a known instability of dAC, with a reported $t_{1/2}$=10 h at physiological temperature and pH[9]. Hydrolytic cleavage of the carbonate bond depends on the pH of the solution/media and the expression levels of carboxylesterase enzymes by the cancer cell. Briefly, a hydrolytic attack at the carbonate bond (green) releases the free phototag, leaving the 3′-carboxylated derivative of dAC that eventually gets converted to dAC via decarboxylation.

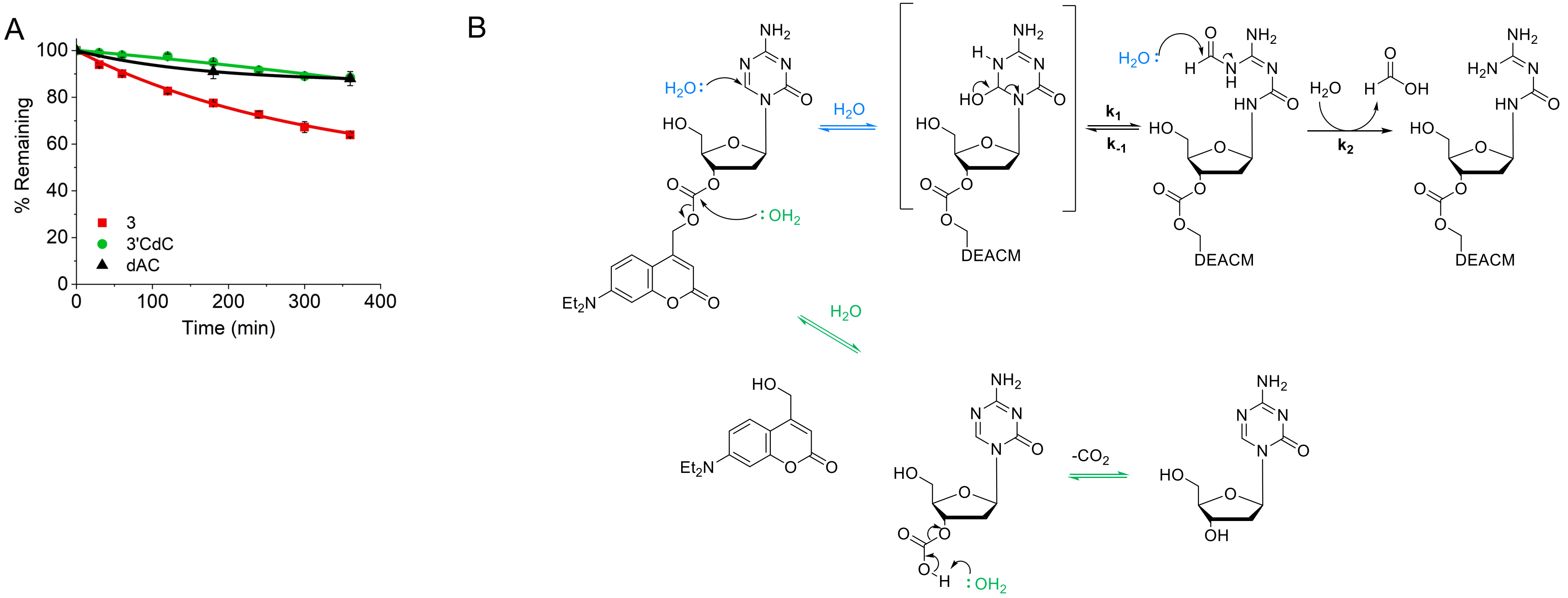

Figure S6. Control of cellular DNA methylation levels in cells. Methylation levels in SaOS2 and T24 cell lines for treatment with dAC (A), compound **3** with light (B), and compound 3 without illumination (C). The concentration of 3 and dAC are indicated. The levels of 5mC were quantified via LCMS and are expressed as a percentage of total cytosines and represent biological triplicates.

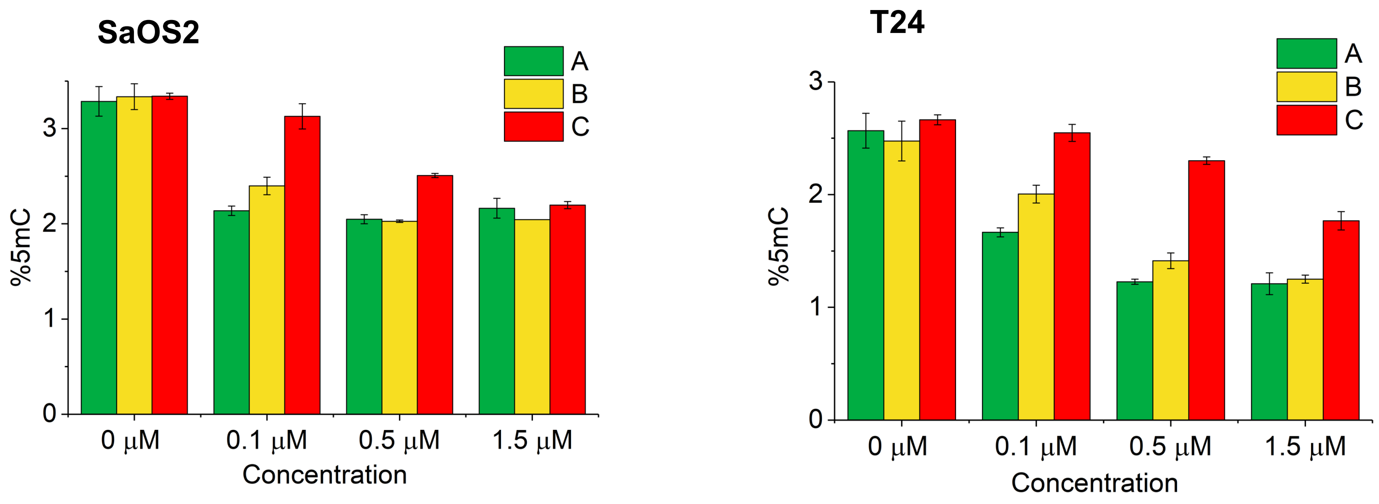

Figure S7. Compound 3 is not phosphorylated by deoxycytidine kinase (dCK). Using the PRECICE® dCK Phosphorylation Screening Assay, kinetic absorbance traces were recorded at λ = 340 nm in the presence of nucleoside analogues, in the dark (black) and following 60 min light exposure at λ = 365 nm (red). (a) When run in standard reaction buffer, the substrate deoxyinosine (dIR) is phosphorylated by dCK. dAC (0.1 mM) and deoxycytidine (0.1 mM) inhibit dIR phosphorylation by dCK in the dark and under illumination. (b) In the dark, compound **3** (0.1 mM) cannot competitively inhibit dIR phosphorylation by dCK. However, when exposed to light, uncaged dAC is cleaved and inhibits dIR phosphorylation.

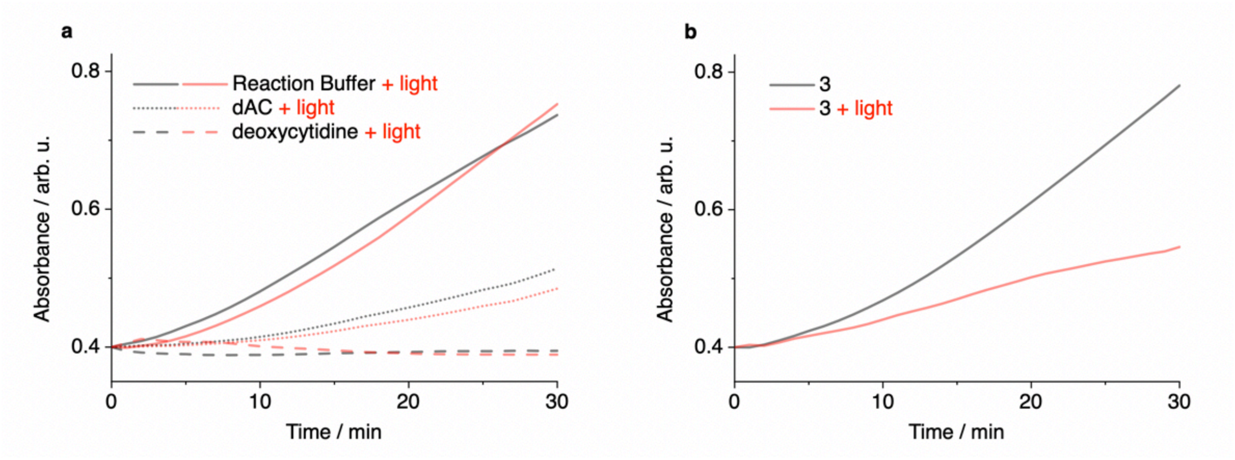

PRECICE® dCK Phosphorylation Screening Assay was used to follow the enzymatic activity of dCK in the presence of nucleoside analogues. The assay is based on the phosphorylation of deoxyinosine (dIR) by dCK enzyme, in the presence of ATP, to form deoxyinosine monophosphate (dIMP) and ADP. dIMP is then oxidized to deoxyxanthosine monophosphate (dXMP) by IMPDH, in the presence of NAD, resulting in the formation of NADH2. The enzymatic activity is monitored by the formation kinetics of NADH2 via absorbance at λ = 340 nm. If the assay is carried out in the presence of a nucleoside competitor, the phosphorylation of dIR is inhibited, resulting in a decrease in NADH2 formation and absorbance at λ = 340 nm.

The assay yielded an increase in absorbance at λ = 340 nm, when run in the reaction buffer (Figure S7A), thereby confirming the phosphorylation of dIR by dCK. In the presence of dAC and deoxycytidine, a decrease in the formation of NADH2 was observed (Figure S7A). The data imply that dAC and deoxycytidine can displace dIR by binding into the active site of dCK. The effect was not compromised when the reaction buffer and nucleoside analogues were irradiated at λ = 365 nm for 60 min prior to absorbance measurements.

When the assay was carried out in the presence of compound 3, NADH2 formation was not affected (Figure S7B). This suggests that **3** does not bind into the active site of dCK. However, when **3** was exposed to light prior to absorbance measurements, a significant decrease in NADH2 formation was recorded. This suggests successful light-induced removal of the photocage from **3**, as well as that uncaged dAC binds into the active site of dCK to competitively inhibit dIR phosphorylation.

Figure S8. Western blot analysis on DNMT1 degradation. (a) SaOS-2 and (b) T24 cells were treated with compound 3 and dAC at concentrations 0.1, 0.5 and 1.5 µM and were either irradiated (light) or kept in the dark (control). Cells were incubated for 24 h followed by cell lysate extraction to measure DNMT1 levels via Western blot analysis. M are non-treated cell extracts.

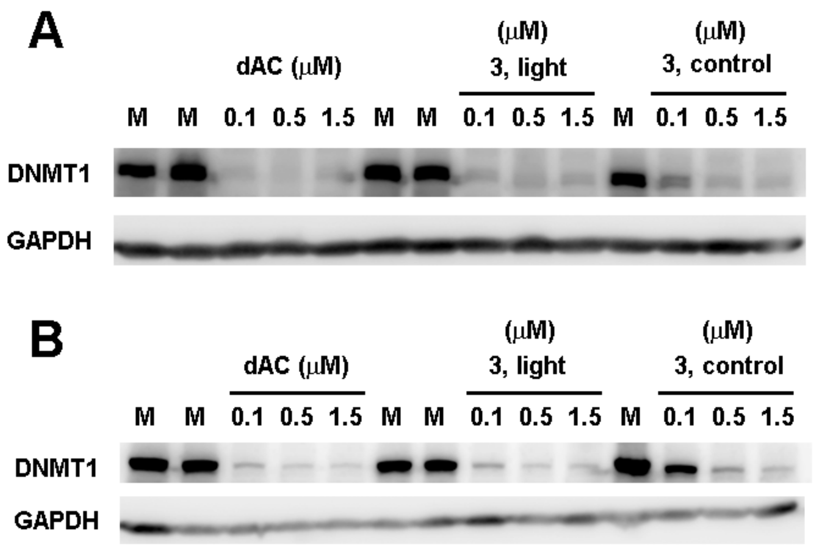

Western blot analyses show the expression levels of DNMT1 and control glyceraldehyde 3-phosphate dehydrogenase (GAPDH) enzymes of SaOS-2 and T24 cells in response to compound **3** with and without illumination, and dAC. Compared to non-treated cell extracts (M), dAC diminished the DNMT1 levels >90% in both cell lines, at all concentrations, as expected. An anticipated effect was also found for compound **3** at a concentration of 0.1 µM. In the absence of light, the DNMT1 levels were higher than in the presence of light, thereby underscoring that light uncaged **3** and reactivated the bioactivity of dAC. As the band for 3-treated yet non-illumined SaOS-2 cells was fainter band than control M, a light-independent DNMT1 degradation activity was inferred. At higher concentration treatments, this effect was not observed. Western blot analyses also showed at higher concentrations of **3** degradation of DNMT1 in the absence of light, which is in line with the observed demethylation effect of **3** at higher concentrations in the absence of light.

Figure S9. ^{1}H-NMR spectra of N-DEACMOC-dAC 1a in DMSO

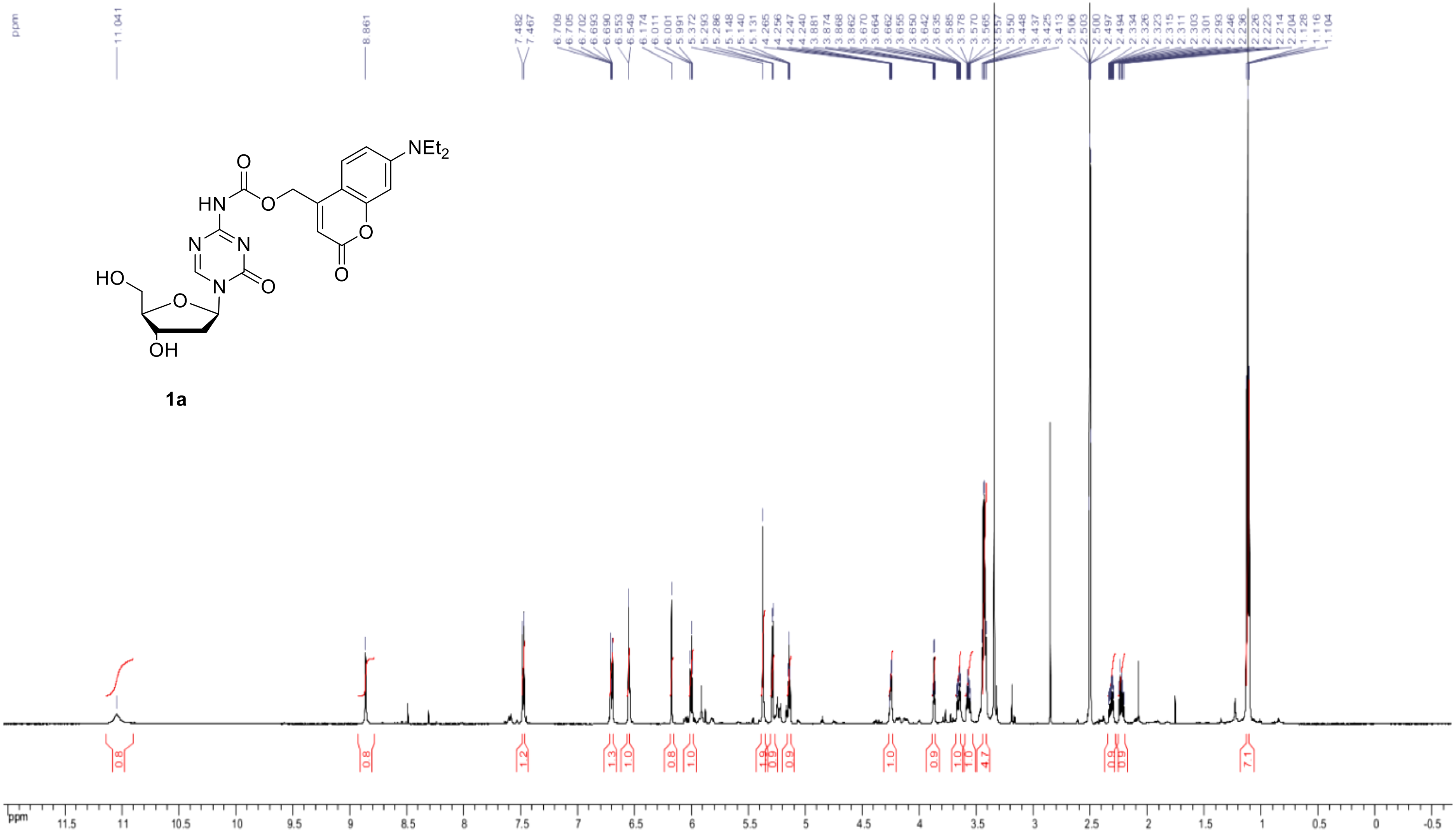

Figure S10. ^{13}C-NMR spectra of N-DEACMOC-dAC 1a in DMSO

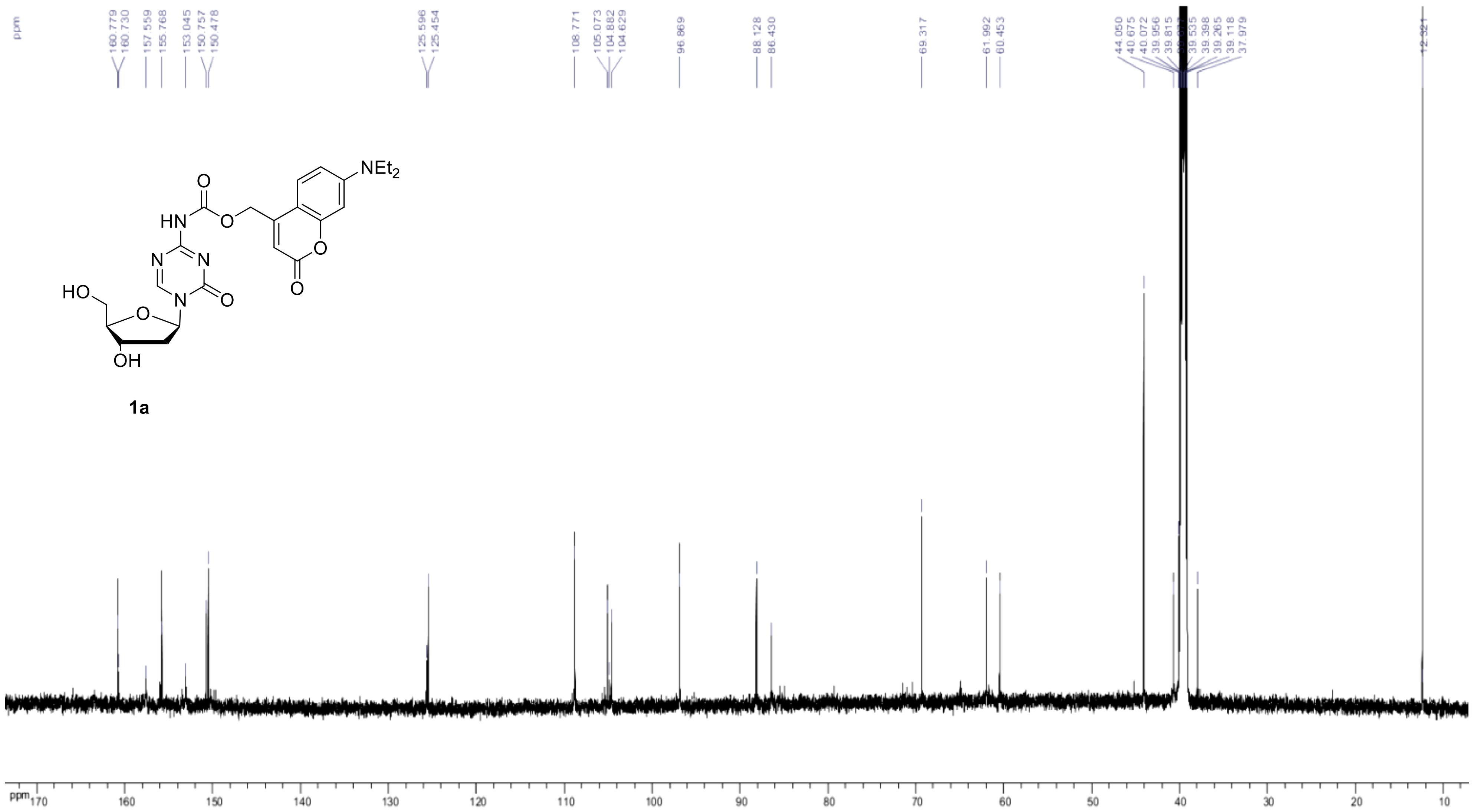

Figure S11. ^{1}**H-NMR spectra of N-NPEOC-dAC 1b in DMSO**

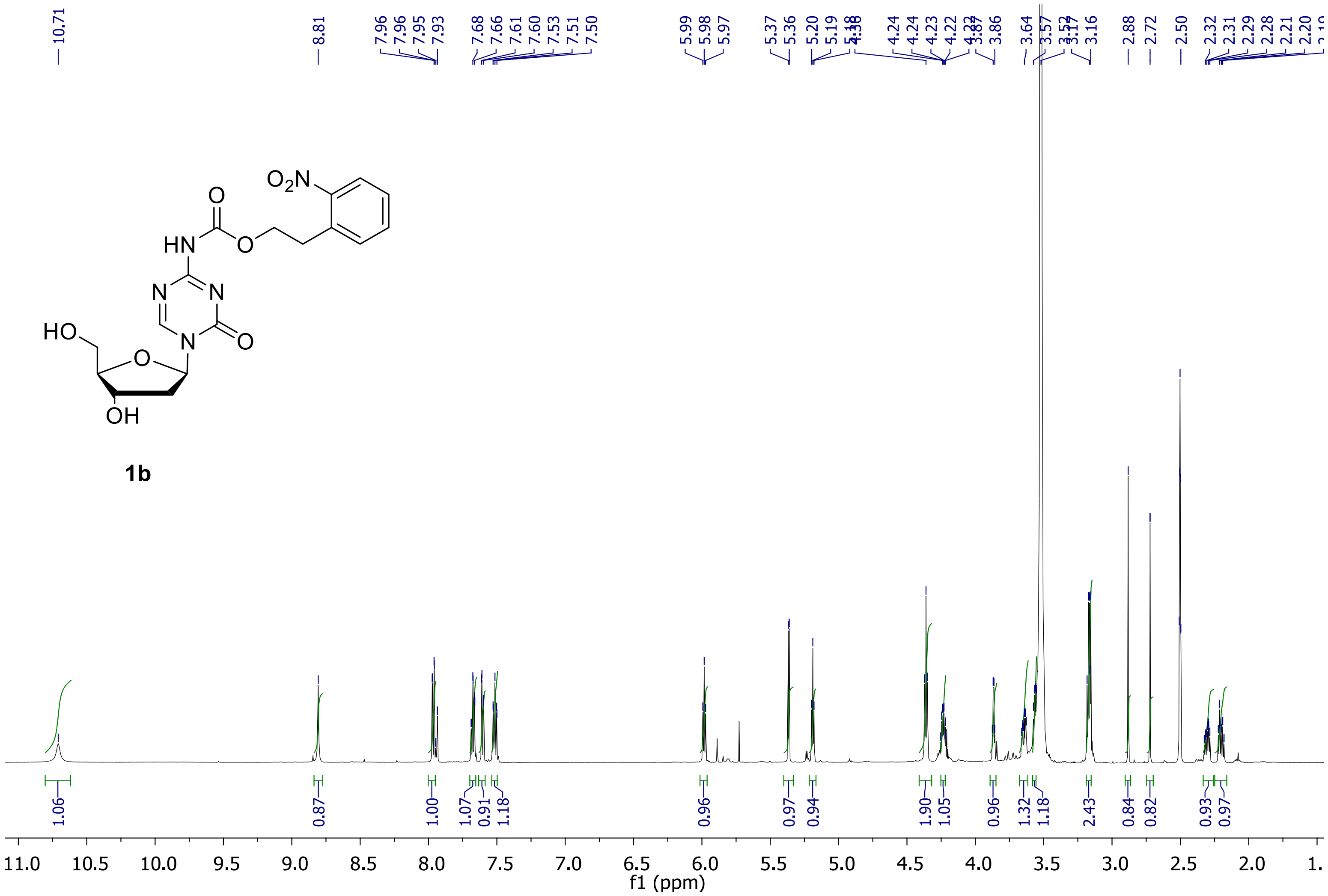

Figure S12. ^{13}C-NMR spectra of N-NPEOC-dAC 1b in DMSO

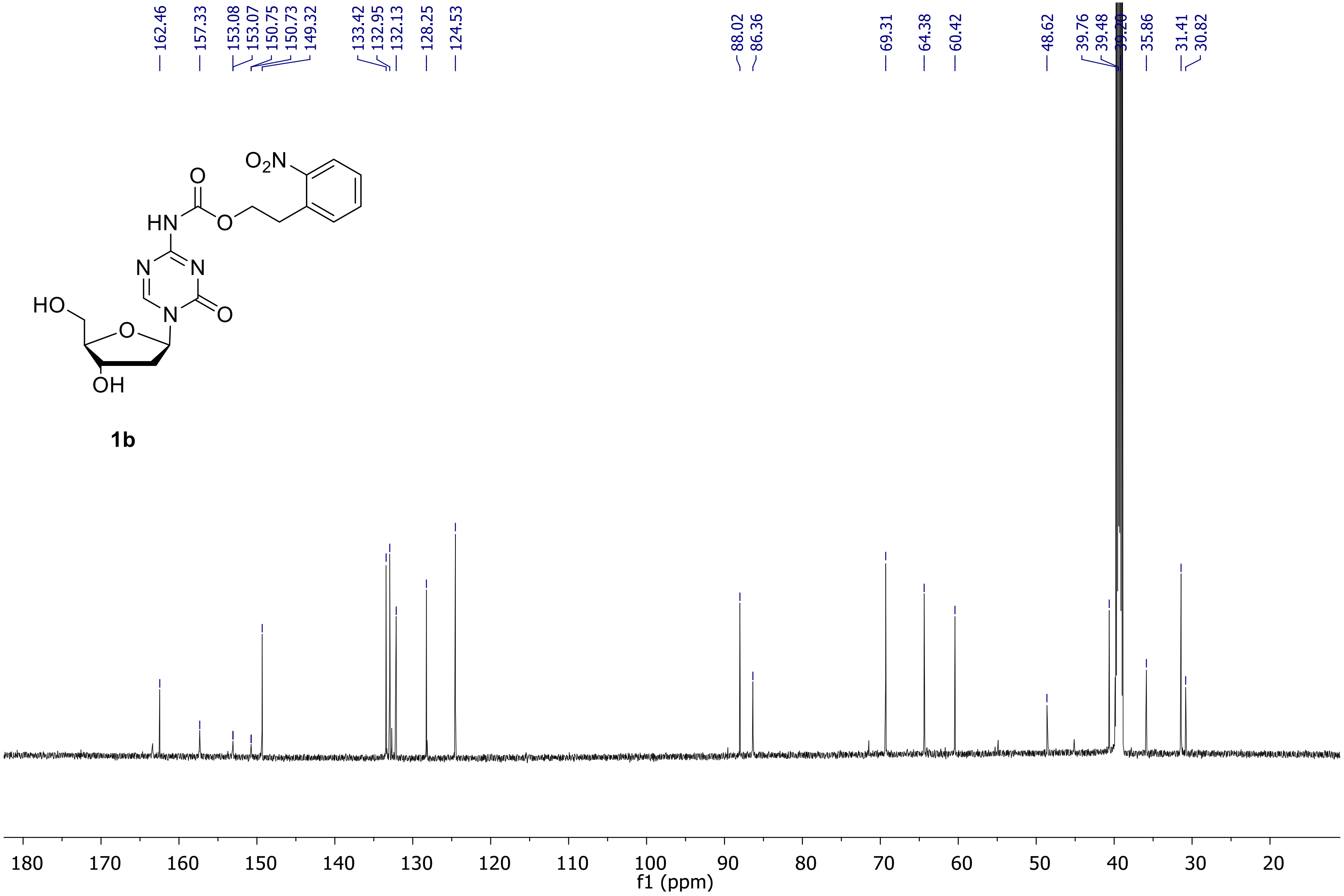

Figure S13. ^{1}H-NMR spectra of N-DMNPEOC-dAC 1c in DMSO

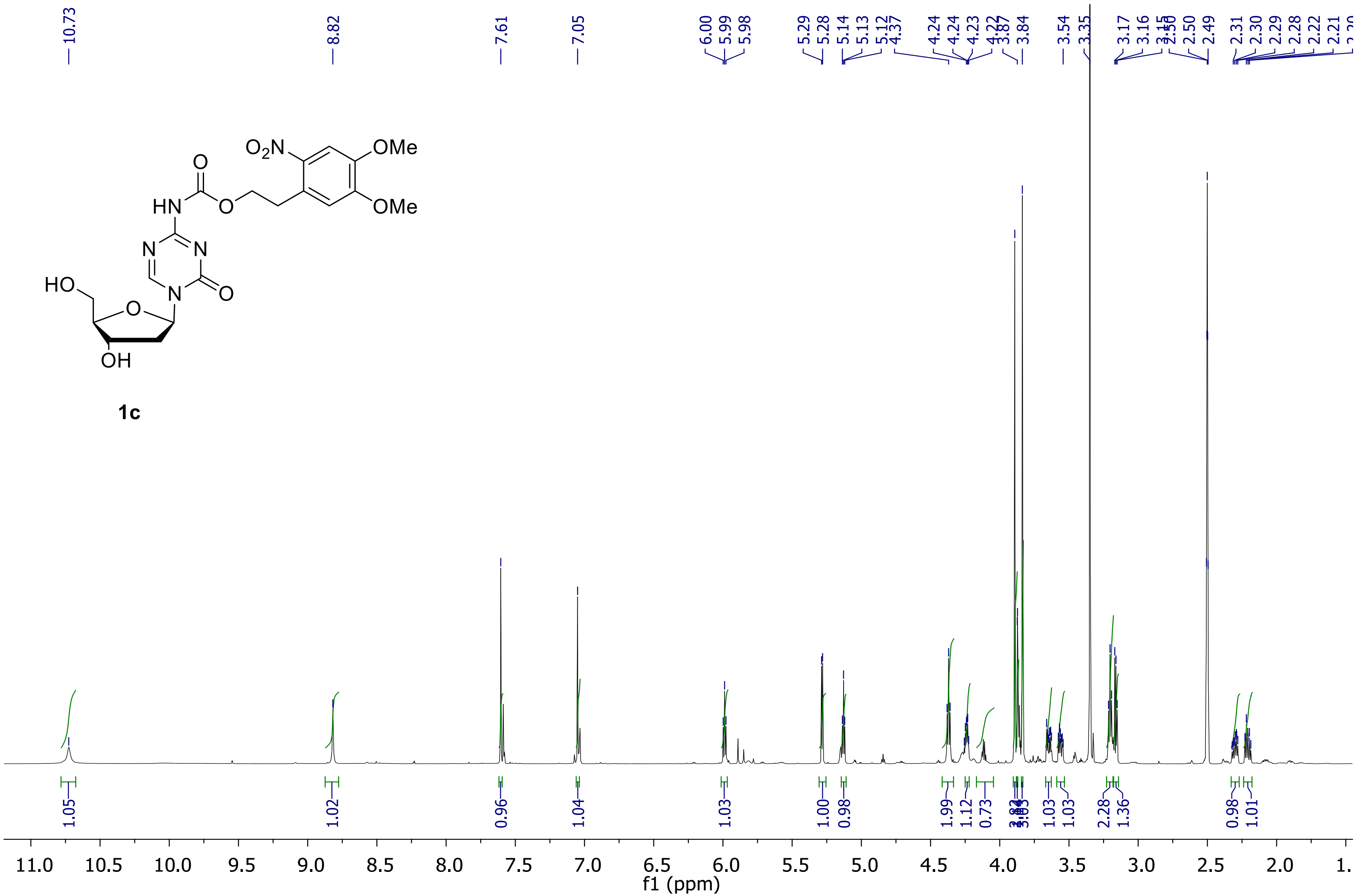

Figure S14. 13C-NMR spectra of N-DMNPEOC-dAC 1c in DMSO

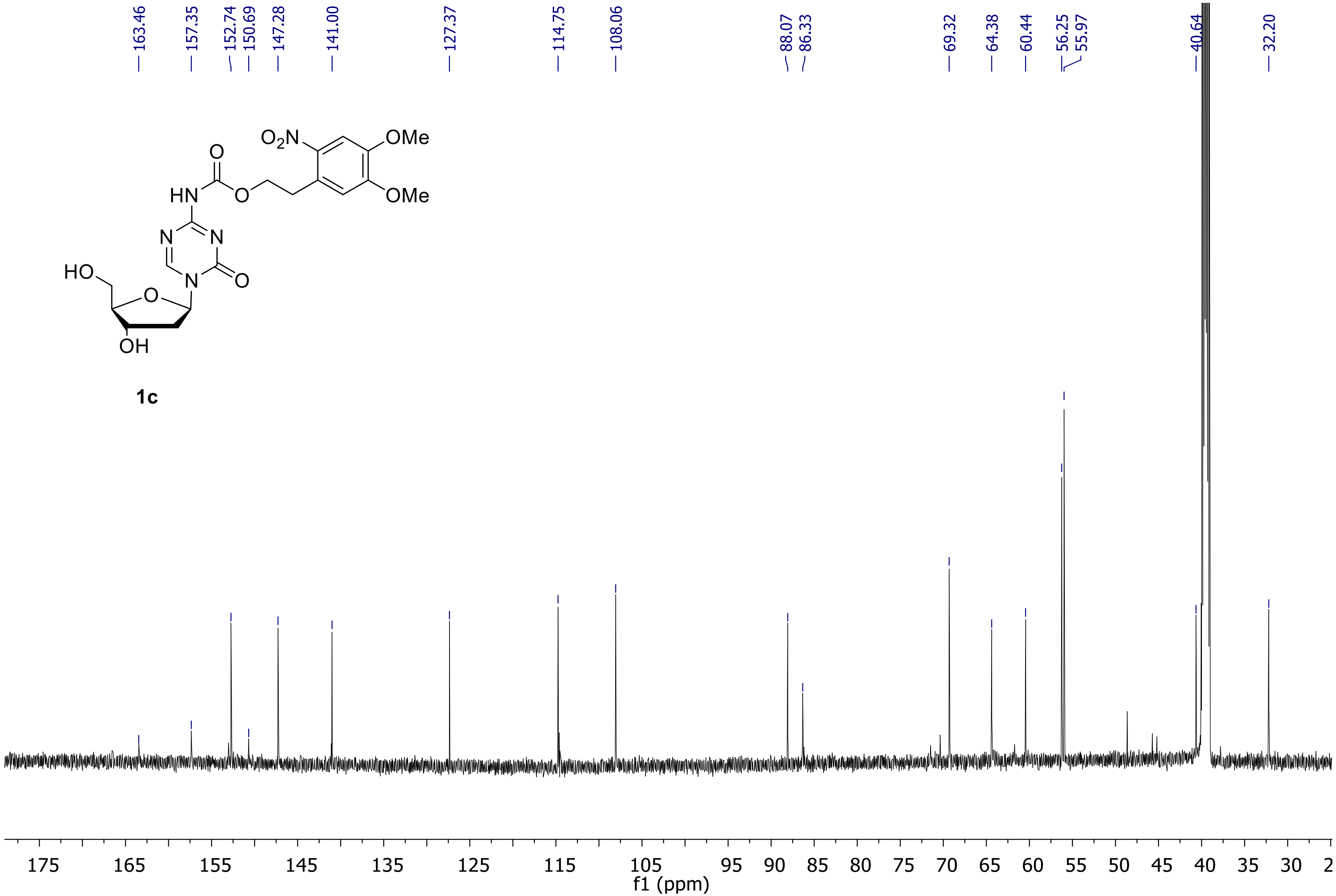

Figure S15. ^{1}H-NMR spectra of bis-NPEOC-AC 1d in DMSO

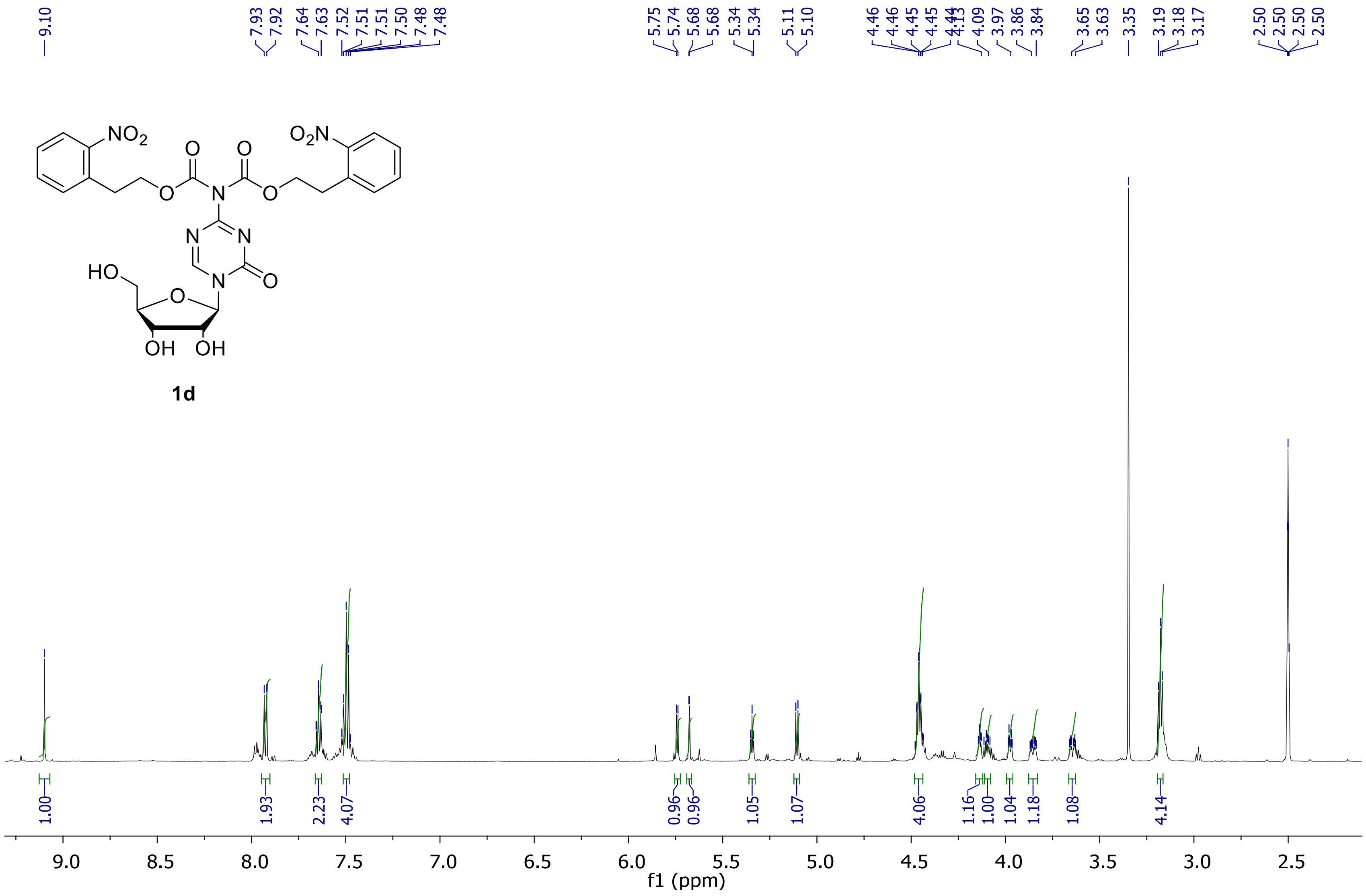

Figure S16. ^{13}C-NMR spectra of bis-NPEOC-AC 1d in DMSO

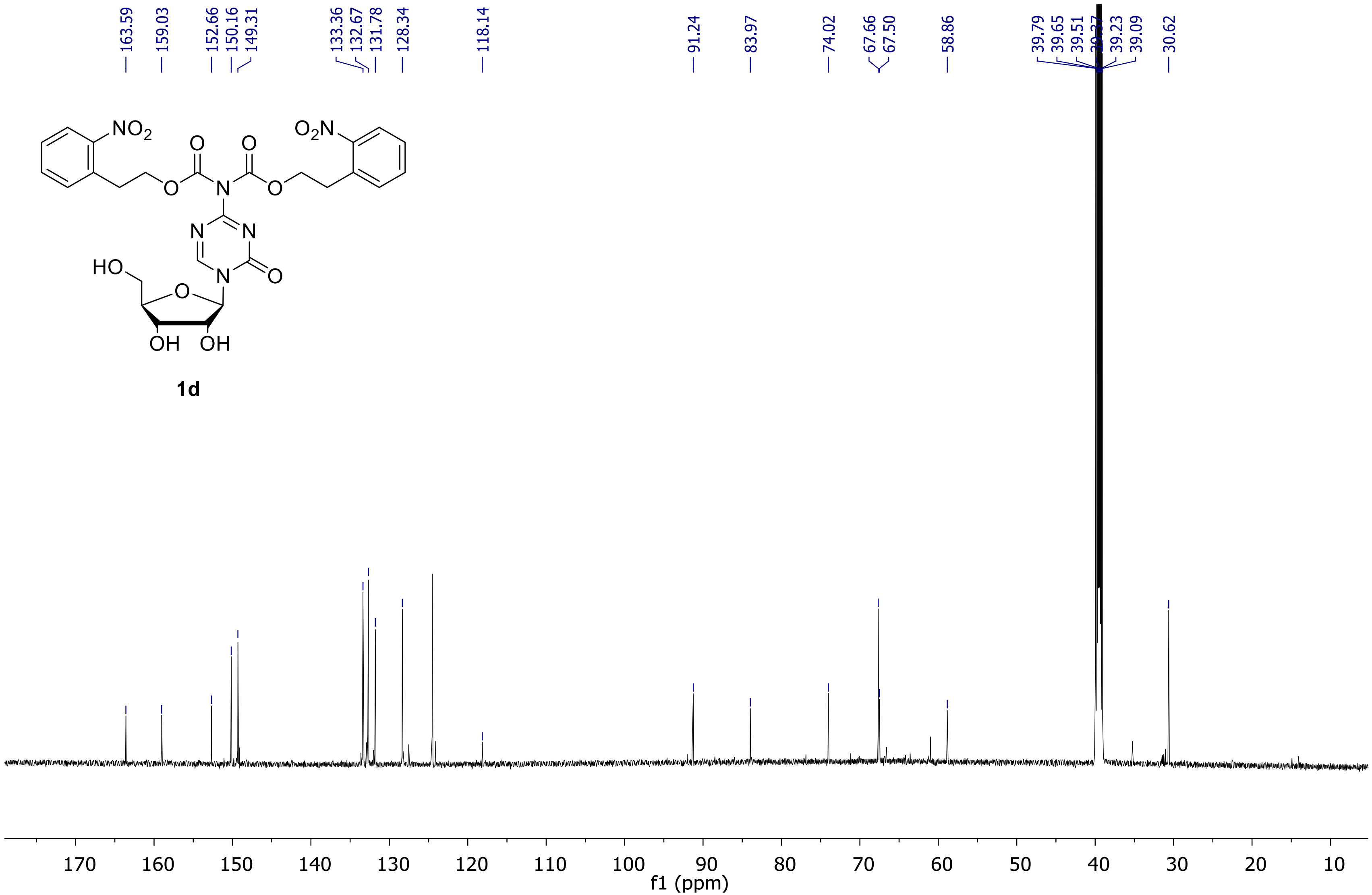

Figure S17. ^{1}H-NMR spectra of 5′-DEACMOC-dAC 2 in DMSO

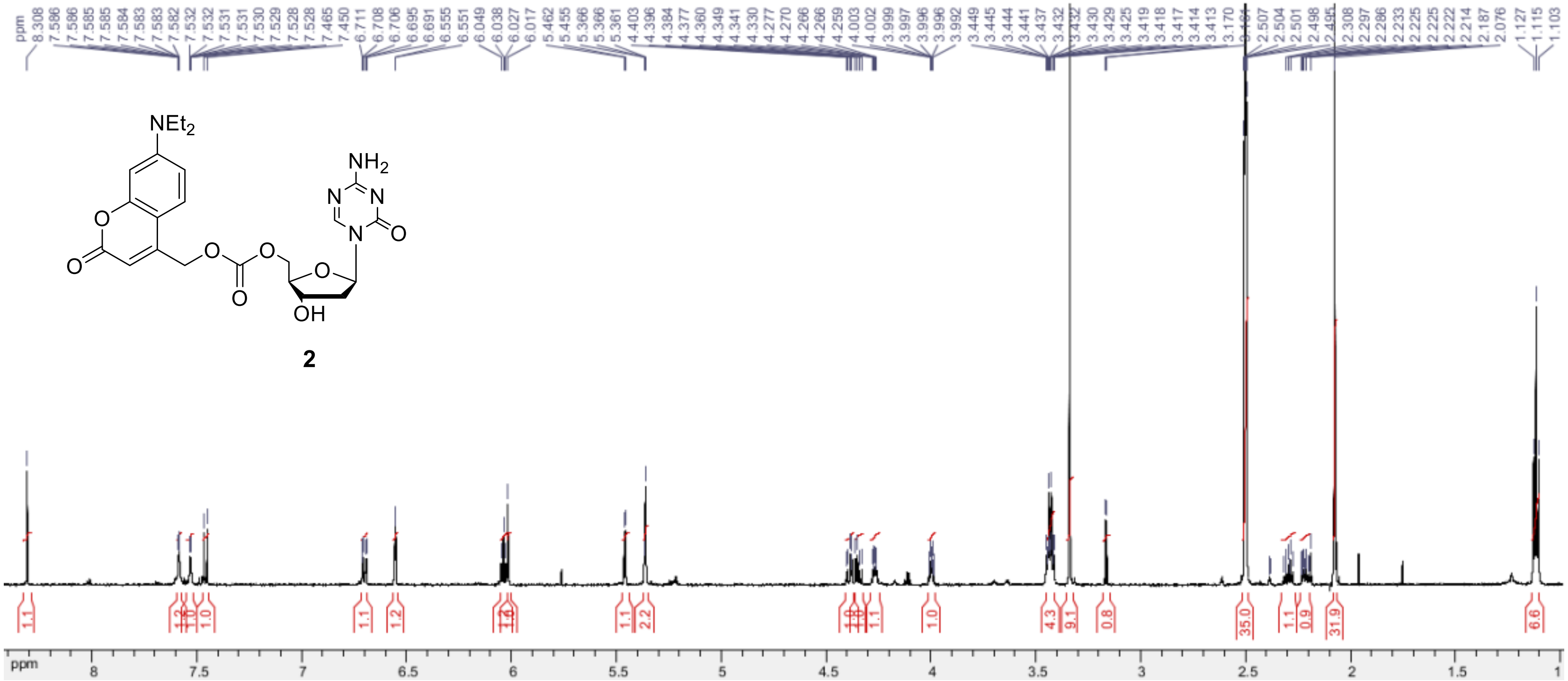

Figure S18. ^{13}C-NMR spectra of 5′-DEACMOC-dAC 2 in DMSO

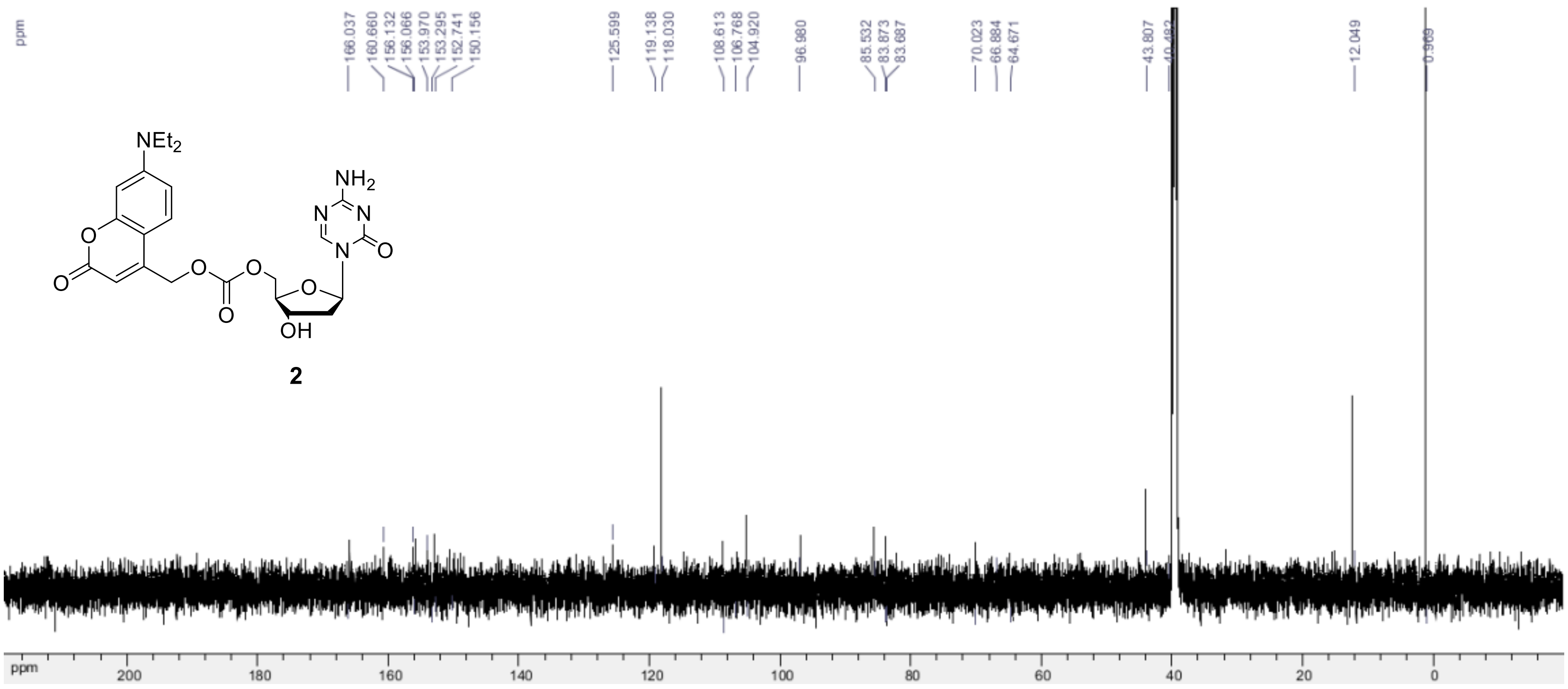

Figure S19. [1]**H-NMR spectra of 3′-DEACMOC-dAC 3 in DMSO**

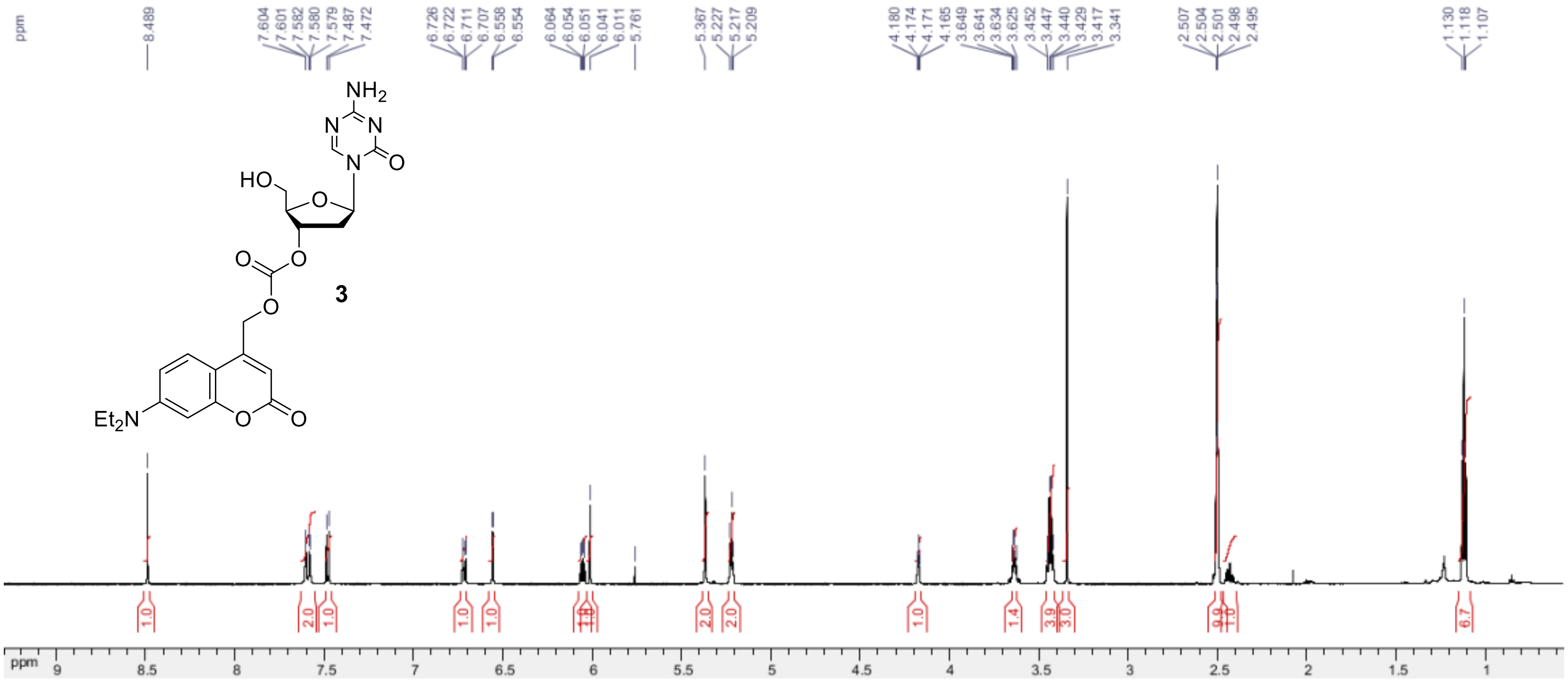

Figure S20. ^{13}**C-NMR spectra of 3′-DEACMOC-dAC 3 in DMSO**

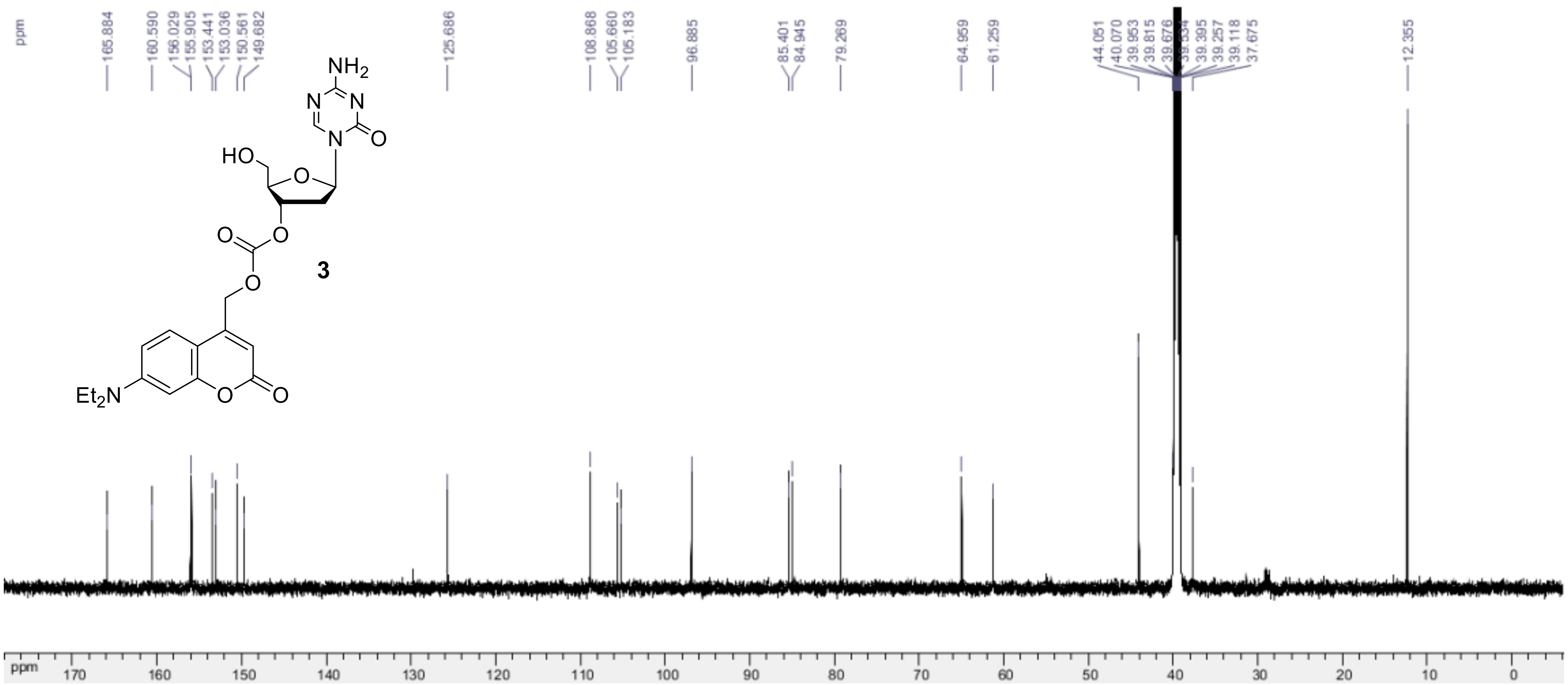

References

1. Rodan, S. B. *et al.* Characterization of a human osteosarcoma cell line (saos-2) with osteoblastic properties. *Cancer Res.* **47,** 4961-4966 (1987).
2. Bubenik, J. *et al.* Established cell line of urinary bladder carcinoma (t24) containing tumour-specific antigen. *Int. J. Cancer* **11,** 765-773 (1973).
3. Kinns, H., Badelt-Lichtblau, H., Egelseer, E. M., Sleytr, U. B. & Howorka, S. Identifying assembly-inhibiting and assembly-tolerant sites in the sbsb s-layer protein from geobacillus stearothermophilus. *J. Mol. Biol.* **395,** 742-753 (2010).
4. Garcia, R. G., Brank, A. S., Christman, J. K., Marquez, V. E. & Eritja, R. Synthesis of oligonucleotide inhibitors of DNA (cytosine-c5) methyltransferase containing 5-azacytosine residues at specific sites. *Antisense Nucleic A.* **11,** 369-378 (2001).
5. Schonleber, R. O., Bendig, J., Hagen, V. & Giese, B. Rapid photolytic release of cytidine 5-diphosphate from a coumarin derivative: A new tool for the investigation of ribonucleotide reductases. *Bioorgan. Med. Chem.* **10,** 97-101 (2002).
6. Nadler, A. *et al.* The fatty acid composition of diacylglycerols determines local signaling patterns. *Angew. Chem. Int. Ed.* **52,** 6330-6334 (2013).
7. Hasan, A. *et al.* Photolabile protecting groups for nucleosides: Synthesis and photodeprotection rates. *Tetrahedron* **53,** 4247-4264 (1997).
8. Swenton, J. S., Bonke, B. R., Chen, C. P. & Chou, C. T. Anodic-oxidation studies of para-methoxyanilides - a general-method for preparation of acylated quinone imine ketals. *J. Org. Chem.* **54,** 51-58 (1989).
9. Rogstad, D. K. *et al.* Chemical decomposition of 5-aza-2'-deoxycytidine (decitabine): Kinetic analyses and identification of products by nmr, hplc, and mass spectrometry. *Chem. Res. Toxicol.* **22,** 1194-1204 (2009).
